# Development of a highly efficient base editing system for *Lactobacilli* to improve probiotics and dissect essential functions

**DOI:** 10.1007/s00253-025-13489-z

**Published:** 2025-04-22

**Authors:** Hitoshi Mitsunobu, Yudai Kita, Yumiko Nambu-Nishida, Shoko Miyazaki, Kensuke Nakajima, Ken-ichiro Taoka, Akihiko Kondo, Keiji Nishida

**Affiliations:** 1 Engineering Biology Research Center, Kobe University, Kobe, Hyogo Japan; 2 Graduate School of Science, Technology and Innovation, Kobe University, Kobe, Hyogo Japan; 3 Bio Palette Co, Ltd, Kobe, Hyogo Japan; 4 RIKEN Center for Sustainable Resource Science, Yokohama, Japan

**Keywords:** Genome editing, Target-AID, CRISPR, *Lactobacilli*, Imidazole propionate, *urdA*

## Abstract

**Abstract:**

*Lactobacilli* play essential roles in the food industry and have a significant potential as probiotics and therapeutic agents. Genomic and genetic information has increasingly accumulated and been linked to their various functions, to which transgenic approaches are being performed to verify crucial genes. In order to reasonably develop more useful strains, beneficial traits need to be introduced into any given strains and enhanced or combined based on such genotype characterization. However, for practical use as probiotics or foods, organisms with transgene are hardly acceptable. Here, we have introduced the base editing Target-AID system specifically for *Lactobacilli*, enabling precise installation of point mutations without donor DNA and at multiple genomic loci simultaneously. *Lactiplantibacillus plantarum* has been successfully engineered to reduce production of imidazole propionate, which has been reported to be associated with type 2 diabetes by impairing glucose tolerance and insulin signaling. Additionally, this system enabled transient knock-out of an essential gene, such as one involved in cell division, resulting in severe filamentous cell phenotype. This demonstrates Target-AID is a promising genetic tool for *Lactobacilli* and can accelerate both applied and fundamental research.

**Key points:**

*• Efficient and multiplexable cytosine base editing established in Lactobacilli.*

*• Edited Lactobacillus reducing imidazole propionate associated with the risk of type 2 diabetes.*

*• Transient knock-out and dissection of an essential gene function.*

**Supplementary Information:**

The online version contains supplementary material available at 10.1007/s00253-025-13489-z.

## Introduction

*Lactobacilli* are Gram-positive, rod-shaped lactic acid bacteria and facultative anaerobes. They constitute an important part of the microbiome in various parts of the human body (Kechagia et al. 2013; Dempsey and Corr 2022) and are utilized in the production of fermented food products such as cheese and yogurt, which are recognized for their positive health effects. Due to their longstanding safe use in human consumption, *Lactobacilli* are generally recognized as safe (GRAS) microorganisms. They have been reported to play roles in improving gut microbiota, modulating immune function, and mitigating stress-induced phycological disorders, prompting their investigation as probiotics and clinical applications (Bravo et al. 2011; Palomar et al. 2014; Huang et al. 2016; Lew et al. 2019; Veiga et al. 2020). Despite their beneficial effects, the genetic and molecular mechanisms underlying these health-promoting properties are yet to be fully understood. In order to utilize *Lactobacilli* as safe and more effective probiotics and therapeutics, rational engineering tools are much needed not only to elucidate the mechanism of action as well as to improve strains with enhanced efficacy (Dempsey and Corr 2022).

CRISPR-Cas9 system has emerged as a powerful tool due to its efficiency and versatility in genetic manipulation (Hill et al. 2014; Mu et al. 2022). Cas9 induces DNA double-strand breaks (DSBs) at specific genomic sites targeted by guide RNA (gRNA), which are then repaired by the host cell’s repair mechanisms. If the repair process is incorrect, a mutation is introduced at the target site. To facilitate precise mutation, donor DNA containing homologous arms and the desired sequence is often co-introduced. However, in many bacterial species, the cytotoxicity associated with the nuclease activity of Cas9 and their poor DSB repair activities often limit its practical application (Standage-Beier et al. 2015; Li et al. 2018; Liu et al. 2019; Arroyo-Olarte et al. 2021). Multiplex editing, where multiple sites are targeted simultaneously, is even more challenging, as it increases toxicity and requires donor DNA at each site, making the process less efficient. In *Lactobacillus* species, CRISPR-Cas9 has been used in combination with recombineering systems that require donor templates for modification (Song et al. 2017; Leenay et al. 2019). To reduce cytotoxicity, nickase variants of Cas9, which induce single-strand breaks, have been employed along with donor DNA for mutation introduction (Song et al. 2017; Goh and Barrangou 2021). A promising alternative to overcome these challenges is base editing, which uses DNA deaminases tethered to nuclease-deficient CRISPR-systems to directly convert bases, avoiding DSBs and mitigating associated cytotoxicity (Rees and Liu 2018; Porto et al. 2020). Base editing also circumvents the need for donor DNA, simplifying the process and enabling more efficient multiplexing. While base editing has shown efficient editing in a variety of bacteria (Banno et al. 2018; Wang et al. 2018a, 2018b; Chen et al. 2018; Liu et al. 2019; Zhang et al. 2020; Luo et al. 2020; Yu et al. 2020; Zhao et al. 2020), its use within Lactobacillus species has so far been limited to *Lactobacillus lactis* (Tian et al. 2022). This highlights the need for more versatile genetic tools applicable to a broader range of *Lactobacillus* species, including those used in probiotic and food applications.

A recent study highlighted increased levels of Imidazole propionate (ImP) in individuals with type 2 diabetes compared to healthy subjects (Koh et al. 2018). ImP is generated from urocanate through histidine degradation pathway, with urocanate reductase (UrdA) from intestinal bacteria playing a crucial role in its synthesis. Additionally, certain *Lactobacillus* strains, which harbor the *urdA* gene, have been reported to be more abundant in individuals with type 2 diabetes. Therefore, disruption of *urdA* gene in probiotic *Lactobacillus* strains could be a valuable strategy in the development of safer probiotics for controlling type 2 diabetes.

The morphology of commensal microbes has been reported to be important for their colonization and stability in the gut ecosystem (Yang et al. 2016), but their genetic background has not been investigated outside of model bacteria, partly due to lack of knowledge of cell morphogenesis and tools to manipulate it in the probiotic microbes. Changes in cell morphology may have significant impact on colonization and host interaction, as longer cells may get stuck and aggregated while smaller cells may be able to sneak into intercellular space. Timing and position of cell division is one of the determinants of cell length and is directed by cell division protein FtsZ, which has been shown to be essential in model strains such as *E. coli* (Beall and Lutkenhaus 1991; Dai and Lutkenhaus 1991; Bi and Lutkenhaus 1991; Ma and Margolin 1999; Sun and Margolin 2004). To dissect its function, conditional mutants have been created to observe filamentous phenotypes. The conditional knock-out strains such as temperature sensitive mutants are typically adopted while obtaining such mutants by traditional mutagenesis is labor-intensive and time-consuming.

Here, we have applied and optimized base editing method for *Lactobacillus* species using the Target-AID system, a cytosine base editor that employs the cytosine deaminase from sea lamprey and uracil glycosylase inhibitor (UGI) (Nishida et al. 2016; Shimatani et al. 2017; Mitsunobu et al. 2017; Banno et al. 2018). The optimized systems achieved almost 100% editing efficiencies in *Lactiplantibacillus plantarum* and *Lactobacillus gasseri* strains and can be multiplexed. This system enables the creation of a strain with improved property for human health and facilitates functional analysis of an essential gene through transient knock-out achieved by introducing specific point mutations similar to natural mutagenesis, without the integration of foreign DNA fragments.

## Materials and methods

### Bacterial strains, plasmids, and media

Table 1 lists all bacterial strains and plasmids used in this study. The molecular cloning was carried out using conventional DNA ligation or Gibson Assembly (Gibson et al. 2009)*.* The promoters and the pIB184-derived region (*repD* and *repE* for replication in *Lactobacilli* and erythromycin resistant gene *ermB*) were synthesized by GenScript (Song et al. 2017). PrimerSTAR Max polymerase (TaKaRa) and TksGflex polymerase (TaKaRa) were used for PCR amplification. Plasmid DNA was extracted using FastGene Plasmid Mini Kit (NIPPON Genetics Co, Ltd.). Restriction enzymes were purchased from New England Biolabs. Ligation high Ver.2 (TOYOBO) was used for ligation reactions. *E. coli* HST08 Premium Competent Cells (TaKaRa) were used for DNA vector amplification and grown in Luria–Bertani (LB) medium at 37 °C with shaking. DNA sequences were confirmed by Sanger sequencing. The complete sequence of pYK06 and the different modules on the other plasmids are shown on Supplementary Notes.

**Table 1:** Bacterial strains and plasmids used in this study

Strain or plasmid	Genotype or characteristic	Reference
Strains		
*Lactiplantibacillus plantarum* WCFS1	Human isolate type strain	ATCC
*Lactobacillus gasseri* ATCC 33323	Human isolate type strain	ATCC
Plasmids		
pYN_Lg4	pIB184 shuttle vector derivative (*repD*, *repE*, and *ermB*), contains Pldh _(Lc)_, tracrRNA, Ppgm _(Sm)_, P23 _(Ll)_, crRNA direct repeat	This work, Addgene 90194
pYK01	12,171 bp; *Lactobacillus*-*E. coli* shuttle vector elements from pYN_Lg4 with PgyrA _(Sm)_-tracr, Pldh _(Sm)_-Target-AID (nCas9-SH3, PmCDA1-SHL, UGI), P23 _(Ll)_-crRNA	This work
pYK02	12,481 bp; *Lactobacillus*-*E. coli* shuttle vector elements from pYN_Lg4 with Pldh _(Lc)_-tracrRNA, Ppgm _(La)_-Target-AID (nCas9-SH3, PmCDA1-SHL, UGI), P23 _(Ll)_-crRNA	This work
pYK03	12,105 bp; *Lactobacillus*-*E. coli* shuttle vector elements from pYN_Lg4 with PgyrA _(Sm)_-tracrRNA, Pldh _(Sm)_-Target-AID (nCas9-SH3, PmCDA1-SHL, UGI), Prrn4b _(Lp)_-crRNA	This work
pYK04	12,415 bp; *Lactobacillus*-*E. coli* shuttle vector elements from pYN_Lg4 with Pldh _(Lc)_-tracrRNA, Ppgm _(La)_-Target-AID (nCas9-SH3, PmCDA1-SHL, UGI), Prrn4b _(Lp)_-crRNA	This work
pYK05	12,246 bp; *Lactobacillus*-*E. coli* shuttle vector elements from pYN_Lg4 with Prrn3a _(Lp)_-tracrRNA-rrnA _(Ls)_ TT, Ppgm _(La)_-Target-AID (nCas9-SH3, PmCDA1-SHL, UGI), P23 _(Ll)_-crRNA	This work
pYK06	12,459 bp; *Lactobacillus*-*E. coli* shuttle vector elements from pYN_Lg4 with Pldh _(Lc)_-tracrRNA, Ppgm _(La)_-Target-AID (nCas9-SH3, PmCDA1-SHL, UGI), Prrn4b _(Lp)_-crRNA-rrnB _(Ec)_ TT	This work
pYK07	12,149 bp; *Lactobacillus*-*E. coli* shuttle vector elements from pYN_Lg4 with PgyrA _(Sm)_-tracrRNA, Pldh _(Sm)_-Target-AID (nCas9-SH3, PmCDA1-SHL, UGI), Prrn4b _(Lp)_-crRNA-rrnB _(Ec)_ TT	This work
pYK08	12,224 bp; *Lactobacillus*-*E. coli* shuttle vector elements from pYN_Lg4 with Prrn3a _(Lp)_-tracr-rrnA _(Ls)_ TT, Ppgm _(La)_-Target-AID (nCas9-SH3, PmCDA1-SHL, UGI), Prrn4b _(Lp)_-crRNA-rrnB _(Ec)_ TT	This work
pYK09	12,459 bp; *Lactobacillus*-*E. coli* shuttle vector elements from pYN_Lg4 with Pldh _(Lc)_-tracrRNA, Ppgm _(La)_-Target-AID (dCas9-SH3, PmCDA1-SHL, UGI), Prrn4b _(Lp)_-crRNA-rrnB _(Ec)_ TT	This work
pYK10	12,290 bp; *Lactobacillus*-*E. coli* shuttle vector elements from pYN_Lg4 with Pldh _(Lc)_-tracrRNA, Ppgm _(La)_-Target-AID (nCas9-SH3, PmCDA1-SHL), Prrn4b _(Lp)_-crRNA-rrnB _(Ec)_ TT	This work
pYK11	12,290 bp; *Lactobacillus*-*E. coli* shuttle vector elements from pYN_Lg4 with Pldh _(Lc)_-tracrRNA, Ppgm _(La)_-Target-AID (dCas9-SH3, PmCDA1-SHL), Prrn4b _(Lp)_-crRNA-rrnB _(Ec)_ TT	This work
pYK12	12,459 bp; *Lactobacillus*-*E. coli* shuttle vector elements from pYN_Lg4 with Pldh _(Lc)_-tracrRNA, Ppgm _(La)_-Target-AID (nCas9-SH3, PmCDA1-SHL, UGI), Prrn4b _(Lp)_-crRNA-rrnB _(Ec)_ TT	This work

*L. plantarum* WCFS1 and *L. gasseri* ATCC 33323 were cultured in de Man, Rogosa, and Sharpe (MRS) broth statically or MRS agar at 37 °C. The erythromycin (Em) concentrations were 10 µg/ml for *Lactobacilli* and the kanamycin (Km) concentrations were 50 µg/ml for *E. coli*

## Electroporation protocol for *Lactobacillus* strains

Transformation of the plasmids by electroporation of *L. plantarum* and *L. gasseri* was conducted by following the previous study (Leenay et al. 2019). Two ml of the medium was inoculated with a single colony from a plate and incubated statically at 37 °C overnight. One ml of this culture (OD_600_: ~ 5–7) was back-diluted into 25 ml of MRS containing 0.41 M glycine in a 50 ml tube and was incubated statically at 37 °C until the OD_600_ reached approximately 0.85 (approximately 3.5 h). Cells were centrifuged at 2,500 × g (5,040 rpm) for 10 min at 4 °C to pellet by KUBOTA 3740 (AF- 5004 CH fixed angle rotor). The pellets were washed twice with 5 ml of 10 mM MgCl_2_, followed by one wash with 5 ml of SacGly (10% glycerol with 0.5 M sucrose). The cells were then resuspended in 1 ml of SacGly and subjected to centrifugation at 20,000 × g (14,340 rpm) for 1 min by KUBOTA 3740 (AF- 2236 fixed angle rotor). After removing the supernatant, the final pellet was resuspended in 500 µl of SacGly. For electroporation, 60 µl of this suspension and plasmid (2–5 µg) were added to a 1-mm gap cuvette and subjected to electroporation using an ELEPO21 (NEPA GENE) with the following parameters: for poring pulse: voltage, 1250 V; pulse width, 2.5 ms; pulse interval, 50 ms; pulse frequency, 1 time; polarity, + and for transfer pulse: voltage, 150 V; pulse width, 50 ms; pulse interval, 50 ms; pulse frequency, 5 times; polarity, ±. After electroporation, 1 ml of MRS broth was added to the cuvette, transferred the cell suspension to a sterile tube, and incubated statically for recovery overnight at 30 °C. Subsequently, 250 µl of the recovered culture was plated on MRS agar supplemented with erythromycin and incubated at 30 °C until colonies form (2–4 days for *L. plantarum* and 2–7 days for *L. gasseri*).

### Mutational analysis

Isolated colonies from the MRS-agar plate supplemented with erythromycin were inoculated in 500 µl of MRS medium supplemented with erythromycin and allowed to grow at 30℃ overnight. The overnight culture was then spread on MRS-agar plate containing Em. A genomic region encompassing the target sites was PCR-amplified using EmeraldAmp PCR Master Mix (TaKaRa) with appropriate primers listed on Table 2 from randomly selected colonies and the resulting fragments were subjected to Sanger sequencing. The frequency of C-to-T change at the targets from each clone was calculated using EditR, an online base-editing analysis tool (Kluesner et al. 2018). The highest editing frequency was adopted if multiple editable bases present at a target site. The editing frequency was determined as the average frequency of C-to-T changes across four to eight independent biological replicates. The flow of experiments to carry out editing and establish edited strains of *Lactobacillus* species were shown in Supplementary Fig. 7.

**Table 2:** Oligonucleotides used in this study

Oligonucleotide	Sequence (5’– 3’)
Lp_upp fw_yk009	TTTGCAAGCGTTACAAGCAC
Lp_upp rv_yk010	TCAGCCATTCGAACACTGTC
Lp_multiCs fw_yk168	ATGTAACCAATCAGGGGGCG
Lp_multiCs rv_yk169	GGCTAATGTGGTCGATCATG
Lp_urdA fw_yk71	CTAGGCCGTTCACTACAAGC
Lp_urdA rv_yk72	AGGTATGCGTCAATCTGCTG
Lp_ftsZ fw_yk102	ATTAAAGTCATCGGTGTCGG
Lp_ftsZ rv_yk103	CTTGACCATTGTTGACTGGC
Lg_target fw_yk19	GCAGCTGCTGGACATCATT
Lg_target rv_yk73	GACGTAGTGATAACGTCAAG

### Microscopy of FtsZ mutants

To assess the cell morphology of the FtsZ mutants, a colony harboring the mutations was cultured in 500 µl of erythromycin-containing MRS media and incubated overnight at 30 °C. Cell imaging was performed using a KEYENCE BZ- 8000 microscope with a 40 × objective lens.

### Evaluation of ImP production

To measure ImP production, 3 mL of Brain Heart Infusion (BHI) broth was inoculated with an isolated single colony of WT or the *urdA*-edited strain on MRS agar plates, and incubated statically at 37 °C for approximately 15 h until the OD600 reached 1.3–1.4 under the anaerobic condition. Thirty microliters of the 15-h culture were added to 15 mL of BHI supplemented with 10 mM trans-urocanic acid. After incubation at 37 °C statically for 24 h under the anaerobic condition, the supernatant of the cell suspension was collected by centrifugation at 3,260 × *g* for 10 min. Five hundred microliters of each sample was mixed with 100 µL of water/acetonitrile/formic acid mixture (900:100:1) and 500 µL of acetonitrile, and applied to InterSep NH2 cartridge and Oasis PRiME HLB cartridge and the eluent was prepared in 10 mL of water/acetonitrile/formic acid mixture (900:100:1) as samples for LC–MS/MS analysis. LC–MS/MS analyses were performed on the Shimadzu UFLC system (CBM- 20 A/LC- 20 AD/SIL- 30 AC; Kyoto, Japan) with an InterSustain AQ-C18 150 × 2.1 mm, 5 µm column (GL science), coupled to a 4000QTRAP triple quadrupole mass spectrometer (SCIEX, Framingham, MA), with Turbo Ion Spray source with ESI ionization (electrospray ionization) in positive mode. Data were acquired by the software LabSolutions Version 5.118 (Shimadzu).

## Results and discussion

### Development of base editing system in Lactiplantibacillus plantarum

**Fig. 1:**
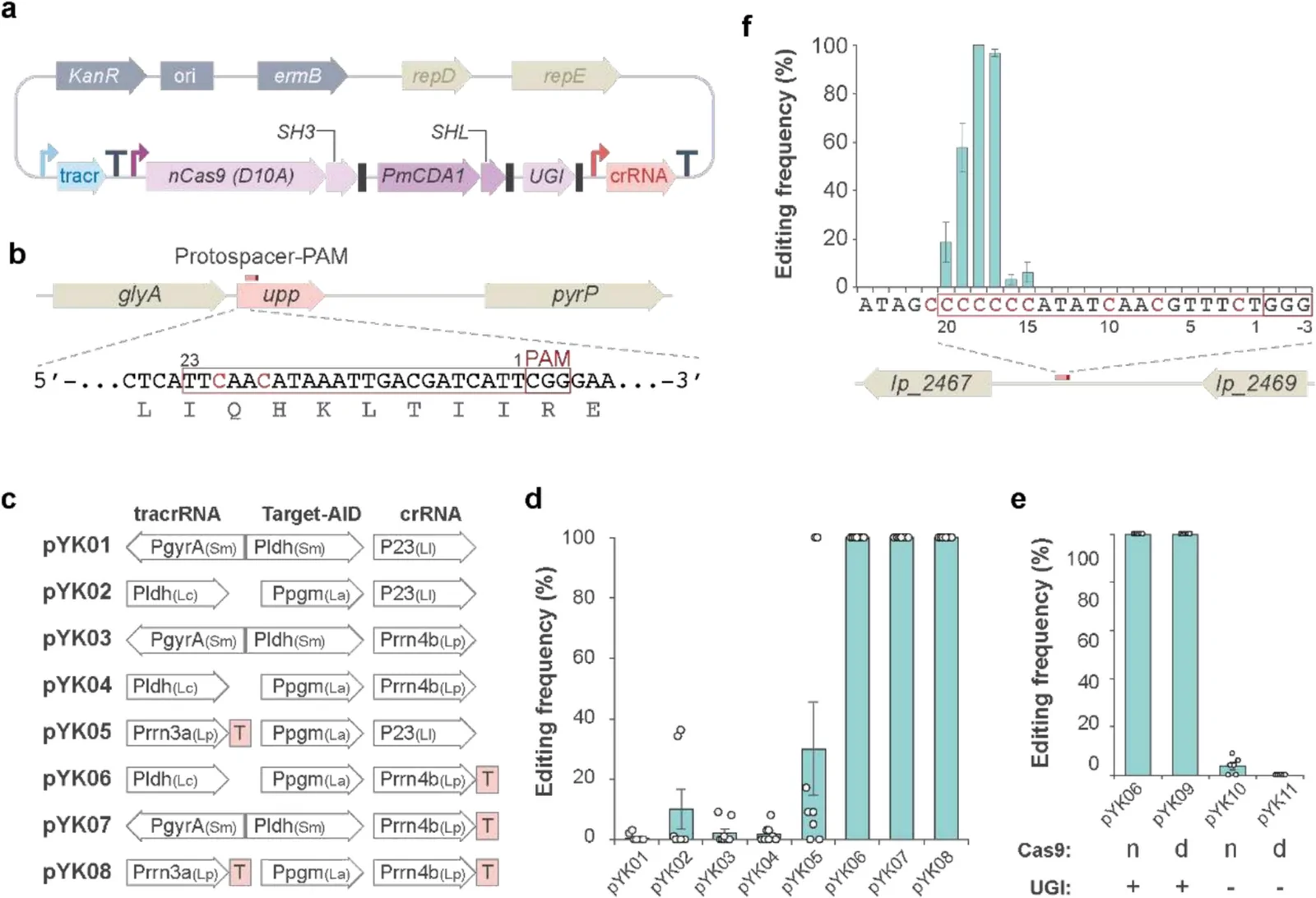
Development of Target-AID base editing system in *L. plantarum*. **a** Target-AID plasmid based on the *Lactobacillus-E. coli* shuttle vector pIB184. TracrRNA and crRNA are expressed as distinct transcripts as dual gRNA system. nCas9, PmCDA1 and UGI are expressed as a single transcript and are translated into separate proteins, as stop codons (black boxes) are placed at the ends of each coding sequence. To mediate the binding of nCas9 and PmCDA1, the SH3 domain and its ligand, SHL, are fused to the respective proteins. The angled arrows represent the promoters. Terminators (T) are placed as necessary. **b** The target sequence design for *upp* gene. The 23 bases of protospacer sequence with PAM are boxed. Cytosines that are subjected to base editing are shown in red. Amino acid sequences are shown at the bottom. **c** The Target-AID plasmid variants with different combination of promoters and terminators. The derived organism for promoters is described in the parentheses. Sm, Ll, Lc, La, and Lp stand for *Streptococcus mutans*, *Lactococcus lactis*, *Lacticaseibacillus paracasei*, *Lactobacillus acidophilus*, and *Lactiplantibacillus plantarum*, respectively. **d** The editing frequencies of each plasmid were determined by sequencing eight randomly selected colonies. The highest frequency among the edited base positions in each colony was plotted as its representative value. The mean editing frequencies are shown as bar graphs, with error bars representing the standard deviation. Independent spectra data for pYK02 and pYK06 are shown in Supplementary Fig. 1. **e** Effect of nCas9, dCas9, and UGI on editing frequency. The bars and error bars represent the mean and standard deviation from six randomly selected colonies, respectively. **f** Editing window of Target-AID in *L. plantarum*. A 20-base target sequence (boxed) containing multiple cytosines (red) was selected from the intergenic region and edited by using pYK6. The mean editing frequencies (bars) at each base position are shown with standard deviation (error bars) from eight randomly selected colonies

To develop an efficient base-editing system in *Lactobacillus* species, we constructed a series of plasmids expressing key components with distinct architectures: nickase Cas9 (D10 A: nCas9), PmCDA1 deaminase, UGI, crRNA, and tracrRNA (Fig. 1a and c–e) with the broad-host-range replication origin from the *Lactobacillus-E. coli* shuttle vector pIB184 used in a wide variety of lactic acid bacteria (Song et al. 2017). The three effector proteins (i.e., nCas9, PmCDA1 and UGI) are expressed as separate proteins by a single promoter in a polycistronic fashion. The crRNA that contains targeting sequence with scaffold was positioned downstream of the effector genes and is driven by a separate promoter. The tracrRNA was expressed under the regulation of either a separate promoter (Fig. 1a) or a bidirectional promoter (Supplementary Information). The bidirectional promoter was located between the nCas9 gene and tracrRNA, expressing the effector proteins and tracrRNA in concert (SI). For the plasmids, pYK05–08, terminators are added downstream of either or both of the tracrRNA and crRNAs. To evaluate the editing efficiency of the constructs, we carried out transformation of *L. plantarum* WCFS1 with these plasmids containing a crRNA targeting the uracil phosphoribosyltransferase (*upp*) gene, a non-essential gene whose loss of function confers resistance to 5-fluorouracil. As shown in previous work with the Target-AID system in other organisms(Nishida et al. 2016; Shimatani et al. 2017; Mitsunobu et al. 2017; Banno et al. 2018), cytosines located within the protospacer of the *upp* gene were in the editing window, approximately 16–20 nucleotides upstream of the 5’ end of the protospacer adjacent motif (PAM: NGG; Fig. 1b). To assess if the system is efficient enough in practical use to obtain a high probability of positive clones, colonies were randomly sampled for sequencing for each construct. With the initial series of constructs pYK01–04, only pYK02 produced colonies coexisting mixed population of the intended C-to-T base-converted mutant and wild type (Fig. 1c, d and SI). However, since the editing frequency of pYK02 was unsatisfactory, we made a second series of constructs to improve the editing efficiency. We found that adding a terminator to the ends of crRNAs significantly improved editing efficiency (Fig. 1d: pYK06–08 and SI). The used terminator *E. coli rrnB* T1 is a well-characterized, efficient terminator that, when transcribed, forms an RNA hairpin and promotes dissociation of the transcription complex (Orosz et al. 1991; Sohn and Kang 2005). It is not dependent on other cellular mechanisms and is therefore presumed to be portable to diverse bacteria. Efficient termination of the transcription may provide intact RNA molecules with longer half-life and/or prevent unintended read-through, which could interfere with the RNA function or expression of downstream components (Curran et al. 2013, 2015; Ren et al. 2025).

In base editing systems, nCas9 is generally preferred to dCas9 as it is more efficient. This is because nicking the opposite strand of a deaminated base inhibits the base excision repair of the deaminated base, facilitating insertion of the mutated base. On the other hand, nCas9 shows somewhat toxicity, especially in bacteria, which can be inferred from the reduced transformation efficiency (Banno et al. 2018; Liu et al. 2020). To see if nCas9 is necessary for efficient base editing in *Lactobacilli*, dCas9 version was tested showing comparable efficiency to nCas9 version as long as it contains UGI (Fig. 1e), which inhibits the initial step of base excision repair. Thus, the dCas9 version can be used in cases such as using a strain exhibiting low transformation efficiency.

### Determination of editing window in L. plantarum

Each base editing system has a unique editing window, meaning that efficient base conversions occur only within a limited range of bases within the target sequence. The editing window for Target-AID has been shown to be at approximately 16–20 nucleotides upstream of the 5’end of the PAM in other organisms (Nishida et al. 2016; Shimatani et al. 2017; Banno et al. 2018). To determine the editing window of pYK06 in *L. plantarum*, we selected a target site containing multiple cytosines at positions 15–21 (distance from the 5’end of the PAM) in an intergenic region (Fig. 1f). Consistent with prior observations, base conversion activity was most pronounced at positions 17–20, with the highest editing efficiency observed at cytosines at positions 17 and 18. In contrast, the cytosine at the position 21 was not converted and the same outcome was observed when the *upp* gene was edited with pYK06. Thus, we concluded that the editing window of this system spans positions 17–20, with lower editing efficiency at position 15 and 16. This limited editing window may constrain the targetable nucleotides, which may be a potential limitation of this system. Other studies in different organisms have shown that different editing systems can generate distinct editing windows and base-change patterns (Nishida et al. 2016; Banno et al. 2018; Wang et al. 2018a; Cheng et al. 2019; Yu et al. 2020; Zhao et al. 2020). Therefore, employing alternative deaminases could broaden the scope of targetable sites, enhancing the potential of this system for genetic manipulation in *Lactobacilli*.

### Expanding targeting scope with NG-Cas9 in L. plantarum

PAM sequence requirement limits the selection of targetable sequence. The original CRISPR-Cas9 system from *Streptococcus pyogenes* requires a PAM sequence of NGG at the 3’end of target sequences. Engineering studies on the Cas9 protein have produced variants with altered PAM requirements, among which NG-Cas9 requires reasonably simplified NG while maintaining substantial editing activity (Nishimasu et al. 2018). By adopting the variant, we intended to develop the Target-AID-NG plasmid for *Lactobacilli*. However, during construction of the requisite plasmid in *E. coli*, the spacer sequence in the crRNA, which encodes target sequence, was found to be frequently mutated and appeared to be self-edited. Due to this effect, we could only obtain a mixed population of plasmids containing the correct spacer sequence and mutated. Although the spacer sequence in the plasmid is followed by GA at its 3’ end and not supposed to be recognized by NG-Cas9, the engineered variant might have ambiguity in the recognition and have self-edited (SI). Using such mixed population of plasmids, we transformed *L. plantarum* to assess its editing capacity at target sites harboring either NGG or NG PAMs (SI). For the Cas9-NG Target-AID system (pYK12, mixed population), we could show editing at the both target sites, albeit with lower efficiency compared to the canonical Target-AID for NGG site (SI), presumably due to impurity of the plasmid. Similar self-targeting effect has been documented for base editing in *E. coli* (Shelake et al. 2023) and plants (Qin et al. 2020), which could be circumvented by using alternative gRNA scaffold sequence starting GCCCC (Qin et al. 2020). Nonetheless, despite these challenges, Target-AID-NG enabled the acquisition of edited clones with practical usability in *Lactobacillus*.

### Efficient multiplex base editing in L. plantarum

**Fig. 2:**
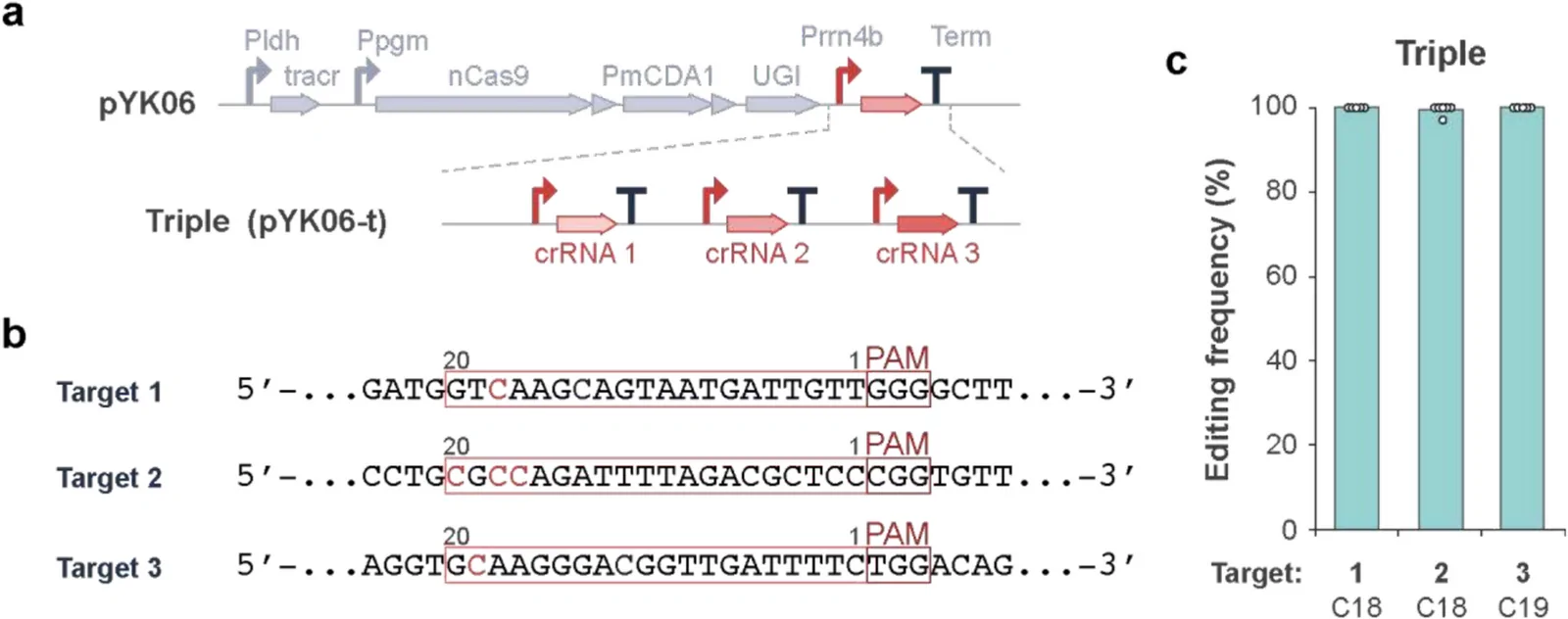
Multiplex Target-AID base editing in *L. plantarum.* **a** Each crRNA expression cassette contains a promoter, a crRNA with a target sequence and a terminator, inserted in tandem in pYK06. **b** Three target sequences are selected from *urdA* gene and editable cytosines are shown in red. **c** The editing frequencies at the three sites by pYK06-t were determined by sequencing six randomly selected colonies. The highest editing frequency at each site were plotted, with the mean editing frequencies shown as bar graphs and error bars representing the standard deviation. The full dataset is available in Supplementary Fig. 3

Multiplex editing using nuclease in bacteria is challenging because it causes severe toxicity and requires donor DNA for each site. As base editing is less toxic and can be multiplexed by multiple crRNA only, we have designed plasmids containing single, dual, or triple crRNA cassettes introduced tandemly in the plasmids (pYK06-s, -d, or -t; Fig. 2a, b and SI). These constructs were transformed in *L. plantarum* with comparable transformation efficiencies. The multiple targeting pYK06-d and pYK06-t achieved highly efficient base conversions (up to 100%) at the all of the targeted sites corresponding to their crRNA cassettes (Fig. 2c and SI), suggesting that the editing process at the one site did not interfere the process at the other sites. Therefore, we could obtain a mutant strain with modifications at three intended genomic sites through one round of editing procedure (transformation and selection), which would be required at least three rounds of procedure with the current recombineering system for the same engineering. This system employs an original dual gRNA consisting of tracrRNA and crRNA, rather than a single-stranded chimeric guide RNA (sgRNA), which means that only the crRNA part is needed for multiplexing, and the shorter repeat sequence length facilitates plasmid construction. In addition, multiple crRNAs can be provided in a manner of CRISPR array, where the spacer sequences are flanked by the crRNA repeat sequences and transcribed under a single promoter regulation, which further decreases the size and complexity of the plasmid.

### Compatibility of the base editing system in Lactobacillus gasseri

**Fig. 3:**
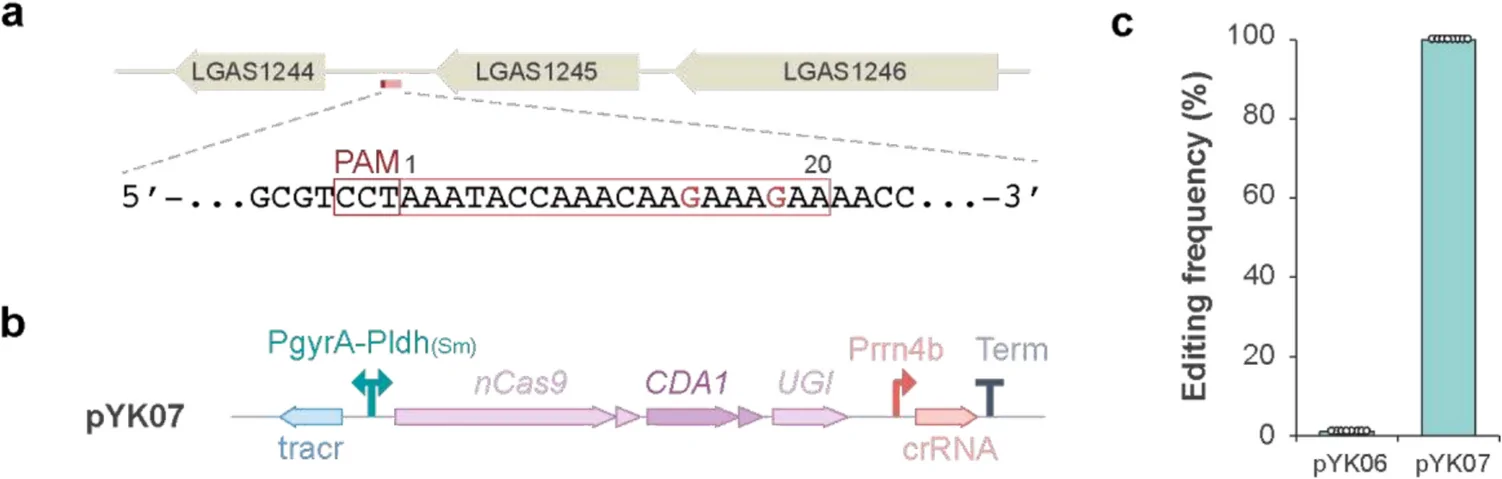
Target-AID base editing in *L. gasseri*. **a** A 20-bases target sequence was selected at intergenic region and the opposite strand is shown with the guanines subjected to editing and shown in red. **b** The architecture of Target-AID in pYK07. The PgyrA-Pldh _(Sm)_ promoter has bidirectional transcriptional activity and drives tracrRNA and effector protein transcript. **c** The editing frequencies of pYK6 and pYK7 in *L. gasseri* were determined by sequencing eight randomly selected colonies. The highest frequency among the edited base positions in each colony was plotted as its representative value. The mean editing frequencies are shown as bar graphs, with error bars representing the standard deviation

The useful variety of *Lactobacillus* species motivated us to investigate whether our base editing system could be adapted to other *Lactobacillus* species. *L. gasseri* ATCC 33323 is commonly found in the human oral and intestinal microbiota, and has a fully sequenced genome (Reuter 2001; Azcarate-Peril et al. 2008). Given that *L. gasseri* is phylogenetically distant from *L. plantarum*, we expected that assessing applicability of our base editing system in *L. gasseri* would strengthen its versatility across diverse *Lactobacillus* species (Qiao et al. 2022). A target sequence was selected in an intergenic region to minimize unexpected effects on cell growth or transformation efficiency (Fig. 3a). Although the transformation efficiency in *L. gasseri* was lower compared to *L. plantarum* under the conditions tested, we were able to obtain transformants with the pYK06 plasmid (SI). However, none of the *L. gasseri* transformants showed detectable base conversion at the target site, likely due to the incompatibility of the promoters from *Lacticaseibacillus paracasei* and *Lactobacillus acidophilus* in *L. gasseri* (0/8 transformants: Fig. 3c). Next, we tested the pYK07 plasmid, which contains a bidirectional PgyrA-Pldh promoter from *Streptococcus mutans*, and had shown high editing efficiency in *L. plantarum* (Fig. 1d). This plasmid resulted in successful base editing in *L. gasseri* (8/8 transformants: Fig. 3b, c, and SI), in contrast to pYK06. These results suggest that PgyrA-ldh is an appropriate promoter for driving expression of the tracrRNA and Target-AID proteins in *L. gasseri*. This underscores the importance of promoter selection in achieving successful genome editing and suggests that the Target-AID system can be adapted to various *Lactobacillus* species including industrially relevant strains, provided plasmid transformation is possible. As conventional genome editing vectors cause higher toxicity that might fail in transformation of industrial and probiotic bacteria, which may benefit more from less toxic base editing system.

### Engineered L. plantarum strain with reduced imidazole propionate production

**Fig. 4:**
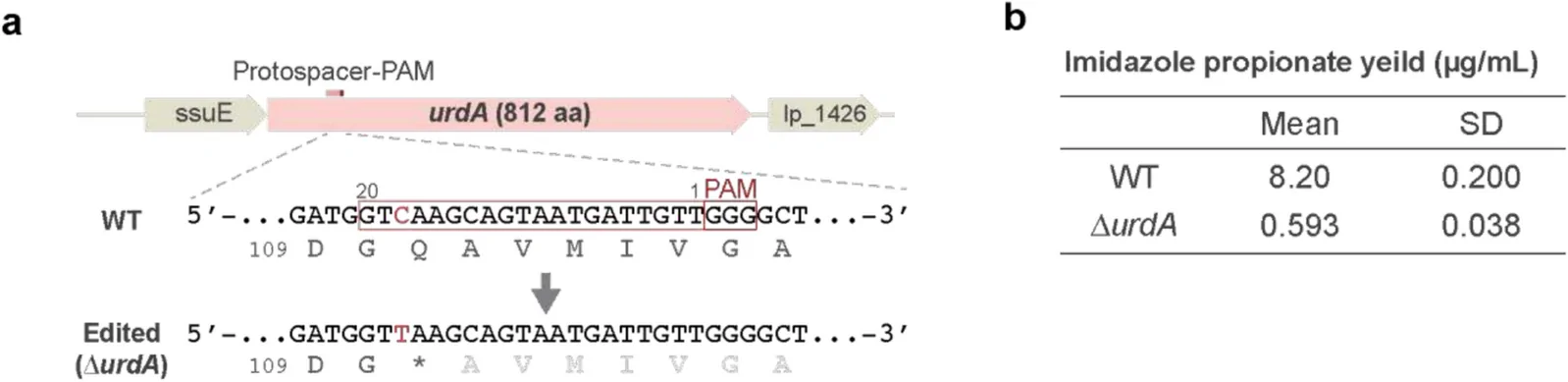
Establishment of *urdA*-edited *L. plantarum* strain with reduced imidazole propionate (ImP) production. **a** Target sequence was selected to install a stop codon at 111 th glutamine (Q) in *urdA* gene. **b** The production of ImP by wild type (WT) and *urdA*-edited strain. Yields were measured in the growth media as described in Materials and Methods

Many of *Lactobacilli* harbor *urdA* gene encoding urocanate reductase that produces imidazole propionate (ImP) from urocanate in histidine utilization pathway (Koh et al. 2018). It has been shown that microbially produced ImP is associated with type 2 diabetes, impairing glucose tolerance and insulin signaling (Koh et al. 2018; Molinaro et al. 2020). To develop a *Lactobacillus* strain that does not produce ImP for use in probiotic and food applications, we designed crRNA targeting the *urdA* gene to introduce a premature stop codon in *L. plantarum* using the pYK06 vector (Fig. 4a). Following transformation, we randomly selected four colonies to obtain nearly 100% editing efficiencies in all cases (SI). Subsequently, to establish transgene-free edited strains, the cells were grown in antibiotic-free medium until the plasmid was lost, which was confirmed by PCR. The disruption of *urdA* did not cause significant growth defects, as the edited strains grew normally compared to the parental strain. We also have confirmed that the *urdA*-disruptant strain is equally capable of fermenting milk with the appropriate supplements required for parental *L plantarum* (Ma et al. 2016). The gene mutation at the edited position was further validated by Sanger sequencing (data not shown). To assess ImP production, supernatants from saturated cultures (OD_600_: ~ 6.0) were analyzed by liquid chromatography-tandem mass spectrometry, revealing a greater than tenfold reduction in ImP levels in the edited strains compared to the wild-type (Fig. 4b). These results demonstrate that *Lactobacilli* can be genetically engineered to reduce ImP production without compromising growth, thus offering a potential approach for developing probiotic and food products that may aid in management of type 2 diabetes. As ImP production is a part of histidine metabolism in *Lactobacilli* that may interact with other pathways, it will be important to assess if reduction of ImP by *urdA* modification may affect original functions of each probiotic to be used. The strains developed with this system essentially maintain the genomic identity of the parental strain, except for the edited loci, which is indistinguishable from the one naturally occurred in quality. Therefore, this system offers a promising approach to add beneficial traits for various *Lactobacillus* strains that already have industrial value, as well as and to accumulate multiple traits in a basic strain that is easy to handle.

### Transient editing and analysis of an essential gene

**Fig. 5:**
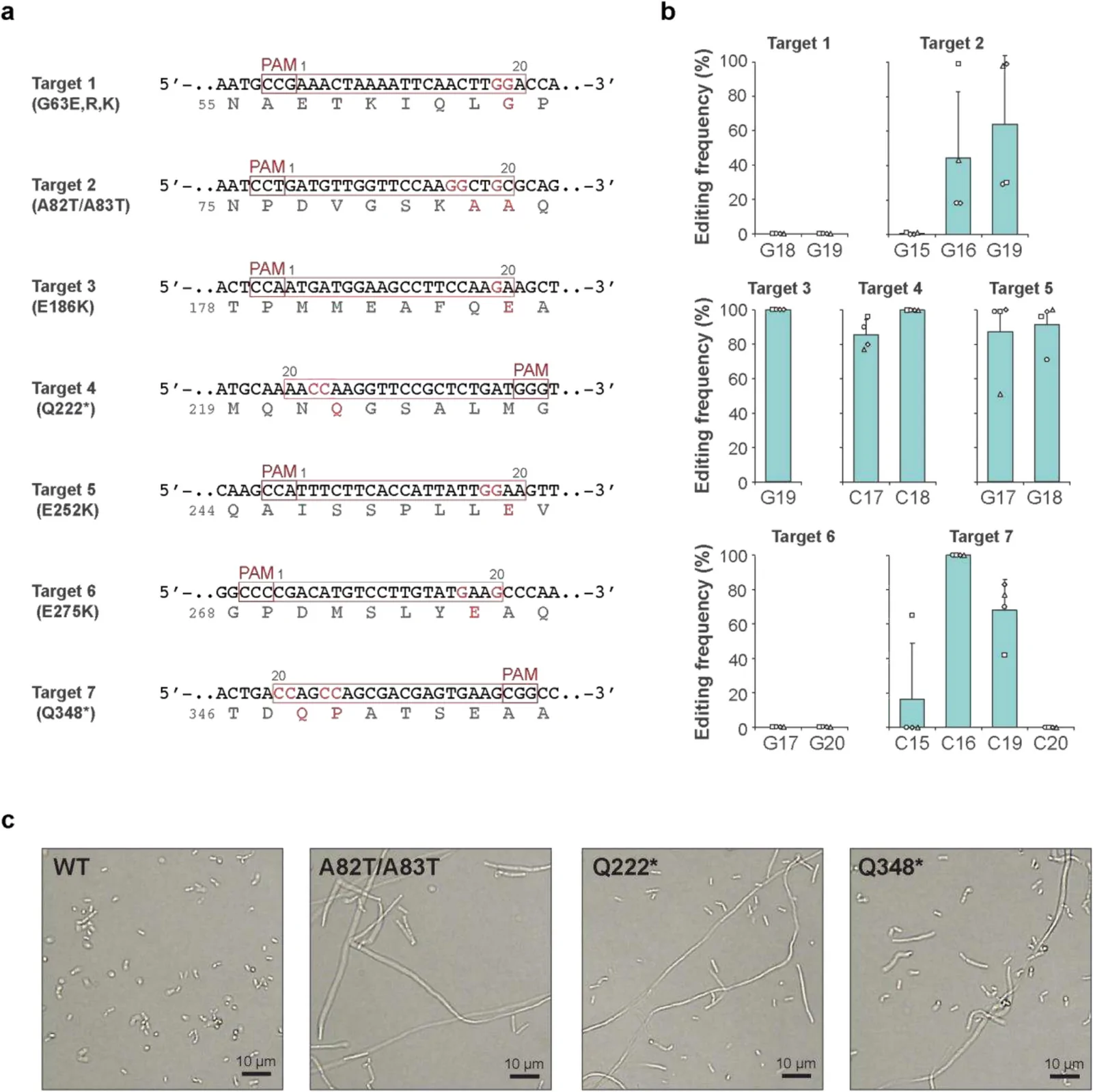
Editing of an essential cell division gene *ftsZ*. **a** Seven target sequences are designed to install amino acid substitutions or premature stop codons in FtsZ. **b** The editing frequencies at editable base positions for each target sequence was determined by sequencing four randomly selected colonies. The mean editing frequencies (bars) at each base position are shown. **c** Optical microscopic images of *wild-type* and representative *ftsZ*-edited cells

The morphology of commensal microbes has been reported to be important for their colonization and stability in the gut ecosystem (Yang et al. 2016), but their genetic background has not been investigated outside of model bacteria. FtsZ is a master regulator of bacterial cell division and its conditional knockout mutants have been shown to have an elongated filamentous cell phenotype (Beall and Lutkenhaus 1991; Dai and Lutkenhaus 1991; Bi and Lutkenhaus 1991; Ma and Margolin 1999; Sun and Margolin 2004). Using pYK06, we designed seven gRNAs targeting the *ftsZ* in *L. plantarum* to introduce amino acid substitutions or stop codons at various positions in the FtsZ coding sequence (Fig. 5a), as its complete loss of function could be lethal. Intended edits were detected in the target 2, 3, 4, 5, and 7 by sequencing of the colonies of obtained transformants (Fig. 5b and SI). We could observe filamentous morphological changes of these mutants in mixed population of edited and wild type cells under optical microscopy (Fig. 5c), indicating that the *ftsZ* function was disturbed. After re-isolation, filamentous cells were still observed as a mixed population of edited and wild-type cells. This suggests that filamentous mutant cells are unlikely to separate and survive, while wild-type cells continue to grow and regenerate a mixed population. We have attempted to obtain stable ftsZ mutant clones devoid of the plasmid on a non-selective plate, but the obtained plasmid-free cells did not retain the mutations, indicating that these mutations cannot be stably inherited over generations in *L. plantarum*. The mixed population indicates a middle state being edited in real-time, allowing transient monitoring of their mutant phenotype. Given that no mutations were found in the target 1 and 6, it is likely that these mutations are more severe and/or cause dominant-negative effects, preventing cells survival even for a short period, while other mutants survive as long as non-mutated proteins remained to work to some extent. This outcome can be an indicator that such mutations are deleterious if they are not obtained at all or are only obtained in such a transient manner. This phenomenon provides a unique approach for transient generation and functional analysis of mutants with lethal or severe growth defective phenotypes like *ftsZ* to dissect essential cellular functions (Beall and Lutkenhaus 1991; Dai and Lutkenhaus 1991; Bi and Lutkenhaus 1991; Ma and Margolin 1999; Sun and Margolin 2004). Conventional methods like random mutagenesis and recombineering would not allow to observe the cells in the process of losing essential gene function. As such, it is difficult to judge if it is because of lethality or experimental problem when the expected mutant cannot be obtained. Therefore, our system serves as a unique tool to investigate important gene functions whose perturbation might cause unexpected severe phenotypes in less characterized organisms including various probiotics. Such investigations are vital for comprehensive understanding of gene-function relationships associated with probiotic activity.

## Conclusion

The Target-AID base editing system enables highly efficient, precise and multiplexable genetic manipulation in *Lactobacilli* by targeted introduction of point mutations at specific loci. These mutations can be rationally designed based on the annotated genomic information or various studies such as transcriptome, proteome and metabolome, as well as genetics on the strain itself or related ones. Traditional breeding and random mutagenesis require intensive screening, which limits the experimentally selectable traits. Multiplexing of traits is more challenging because undesirable mutations also accumulate through each screening. When a useful trait is identified in one strain, our system can transfer it to any other strains and combine with other useful traits easily. For example, researchers can use their own probiotic to apply the concept of base-edited strain with potentially reduced risk for type II diabetes, because base-edited organisms are not subjected to GMO regulation in several major regions as it is indistinguishable to the one naturally occurred in quality (Waltz 2016; Callaway 2018; Spök et al. 2022), which may not be the case for conventional transgenic methods or even conventional CRISPR-Cas9, which is not robust among species and requires template donor DNA to recombine. As the use of genome-editing technologies in food production grows, it is essential to engage in broader ethical and regulatory discussions to ensure their responsible implementation and to foster public trust in these new technologies.

## Supplementary Information

Below is the link to the electronic supplementary material.

Supplementary file1 (PDF 3180 KB)

## Data Availability

No datasets were generated or analysed during the current study.
