# Supplementary material for: Development of a highly efficient base editing system for Lactobacilli to improve probiotics and dissect essential functions
Source: Appl Microbiol Biotechnol. 2025 Apr 22;109:96. doi: 10.1007/s00253-025-13489-z (PMC12014835; doi:10.1007/s00253-025-13489-z)
Supplement: Supplementary file 1 [file 253_2025_13489_MOESM1_ESM.pdf]

Applied Microbiology and Biotechnology

Development of a highly efficient base editing system for *Lactobacilli* to improve probiotics and dissect essential functions

Hitoshi Mitsunobu[a]*, Yudai Kita[b]*, Yumiko Nambu-Nishida[c], Shoko Miyazaki[c], Kensuke Nakajima[c], Ken-ichiro Taoka[a], Akihiko Kondo[a,b,d], Keiji Nishida[a,b]#

[a] Engineering Biology Research Center, Kobe University, Kobe, Hyogo, Japan.
[b] Graduate School of Science, Technology and Innovation, Kobe University, Kobe, Hyogo, Japan.
[c] Bio Palette Co., Ltd, Kobe, Hyogo, Japan
[d] RIKEN Center for Sustainable Resource Science, Yokohama, Japan

Running Head: Base editing by Target-AID in *Lactobacilli*

#Address correspondence to Keiji Nishida, keiji_nishida@people.kobe-u.ac.jp

*Hitoshi Mitsunobu and Yudai Kita contributed equally to this work.

Supplementary Materials

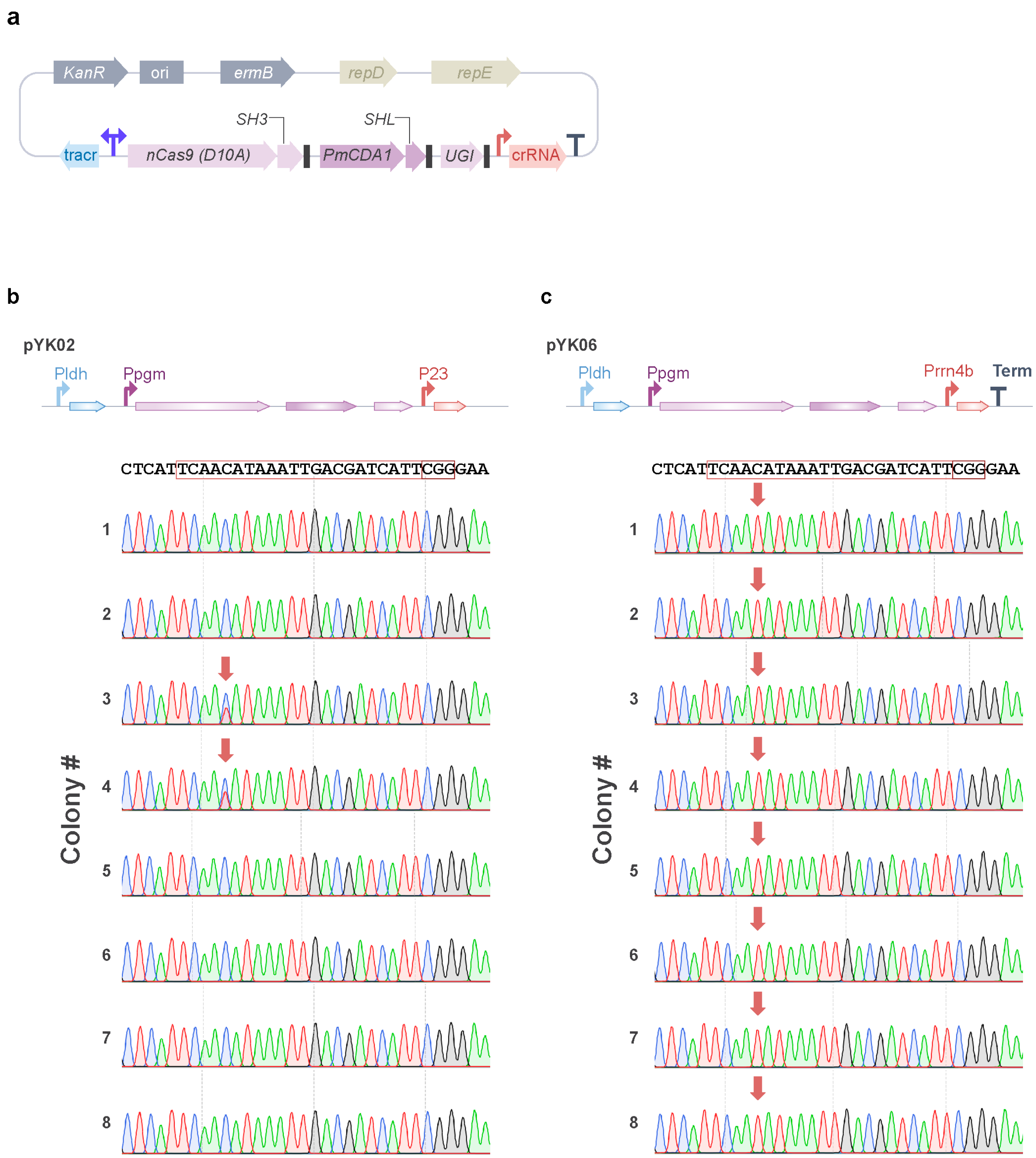

Supplementary Fig. 1

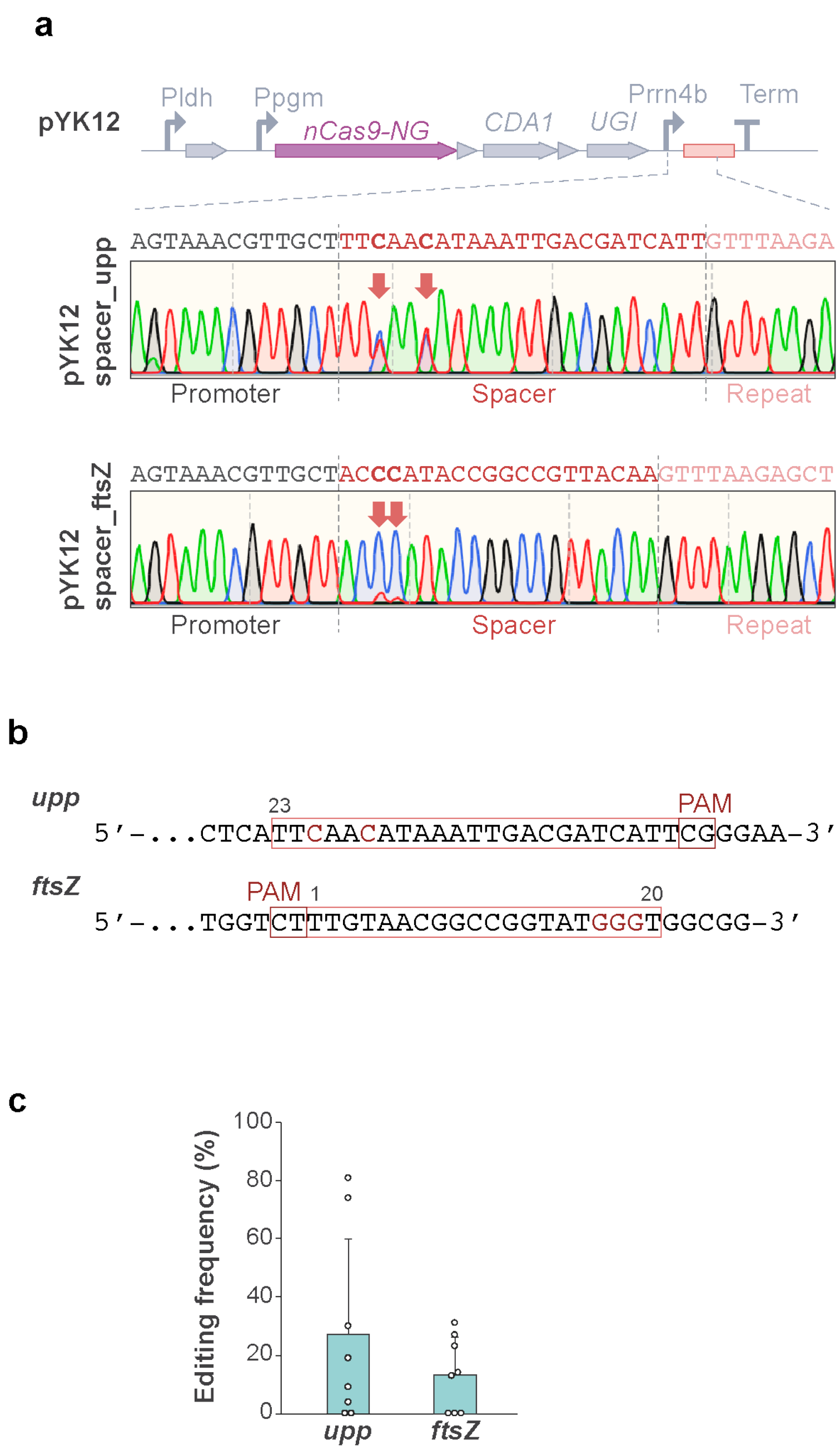

Supplementary Fig. 2

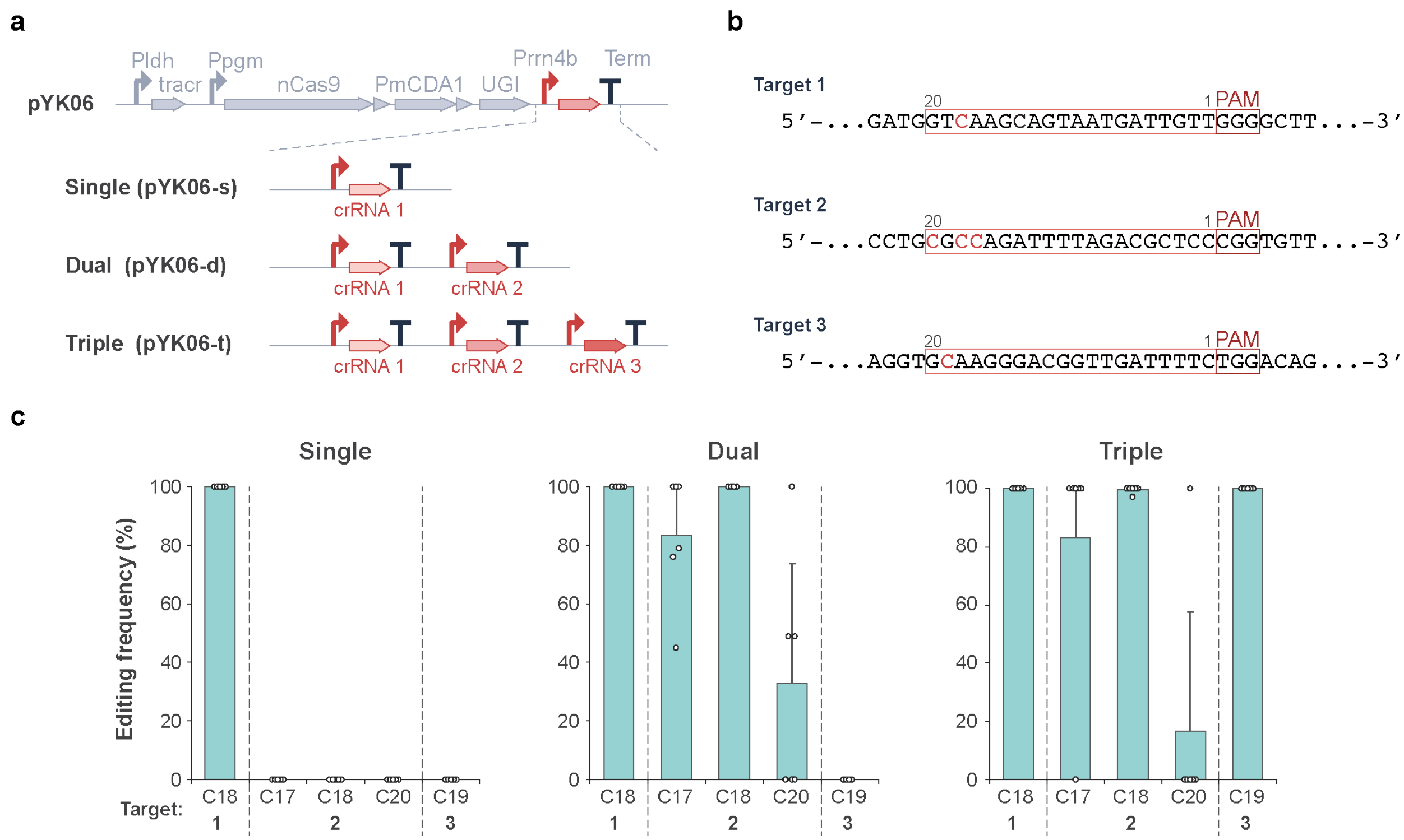

26 **Supplementary Fig. 3**

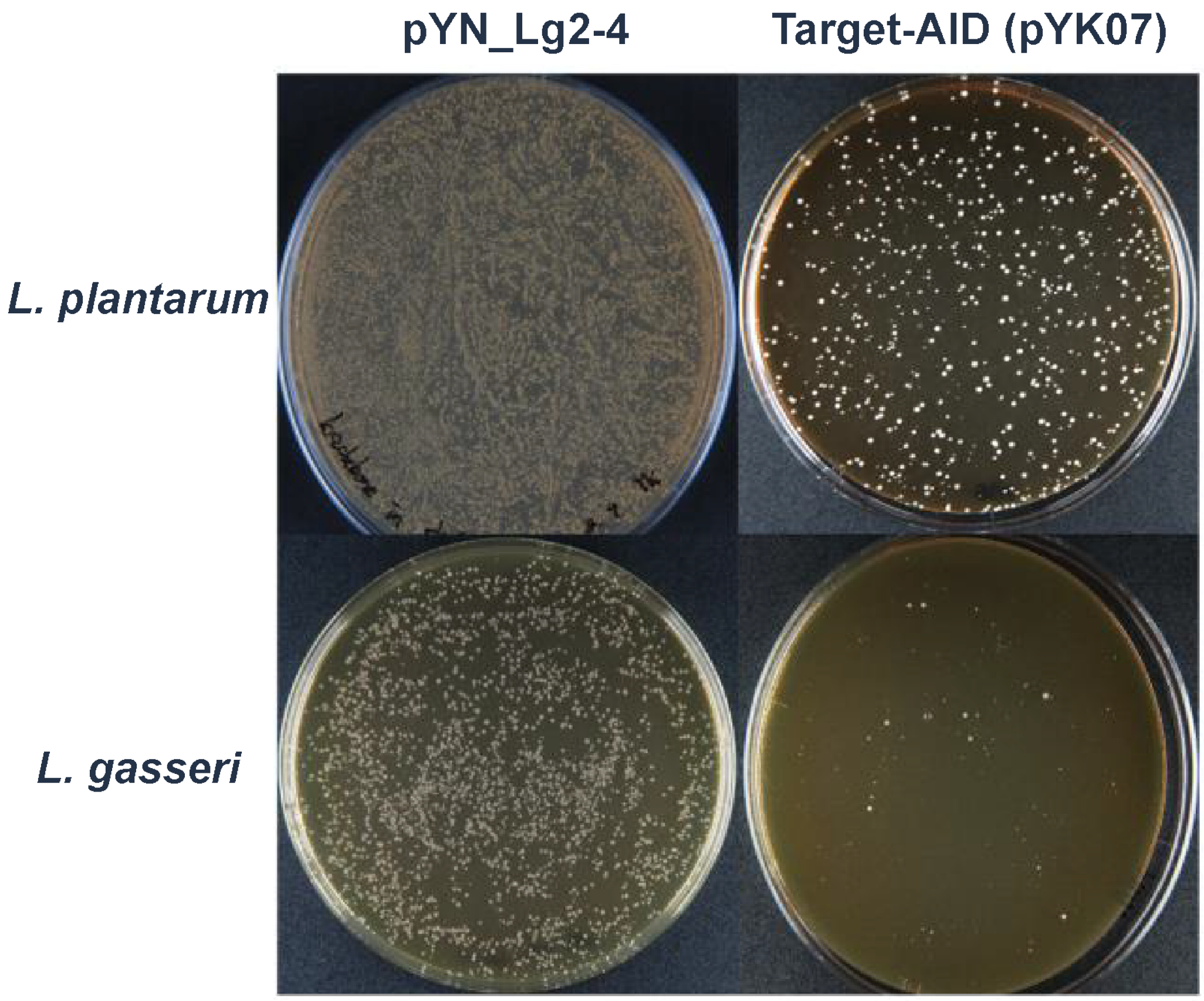

Supplementary Fig. 4

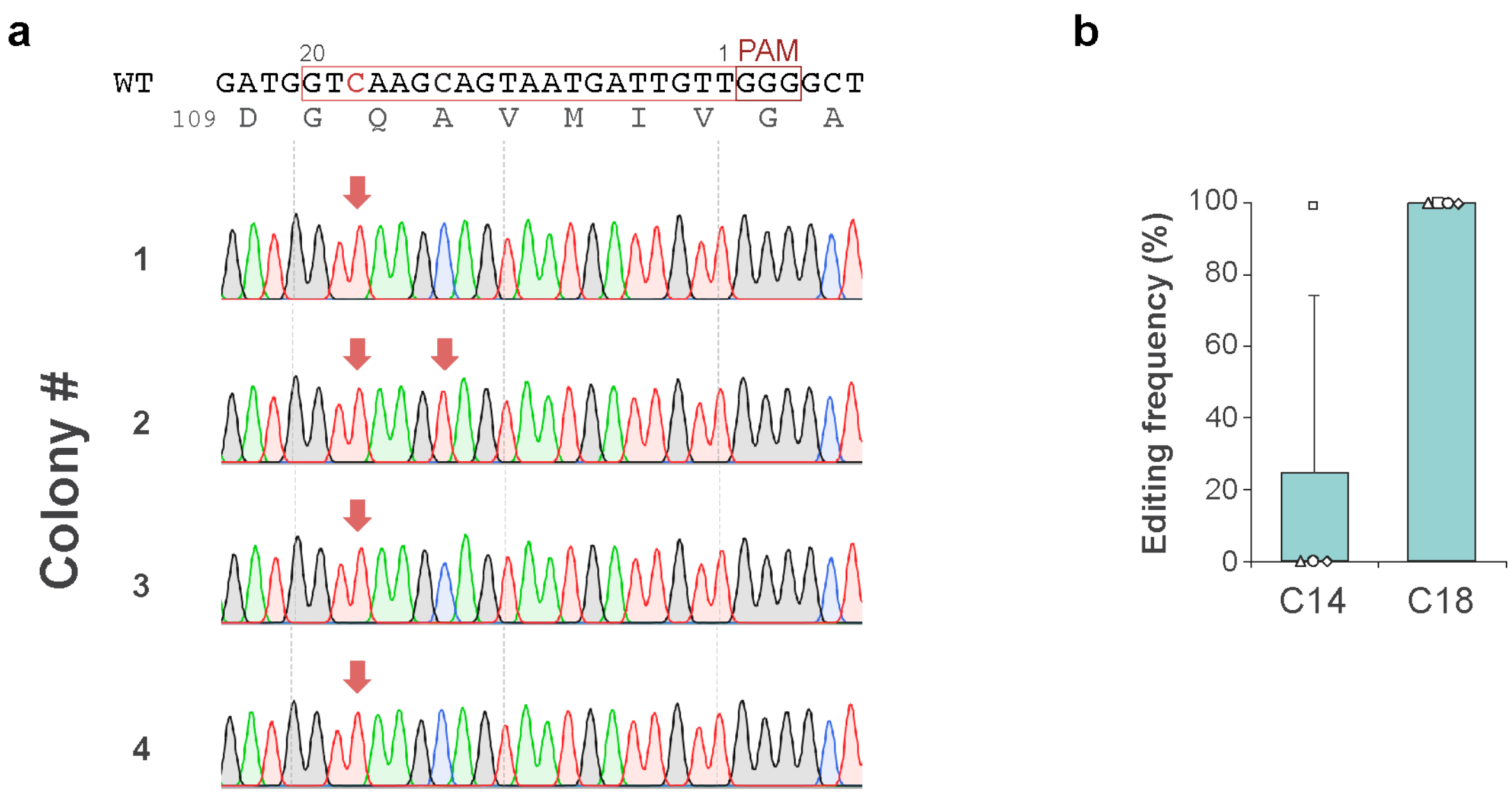

Supplementary Fig. 5

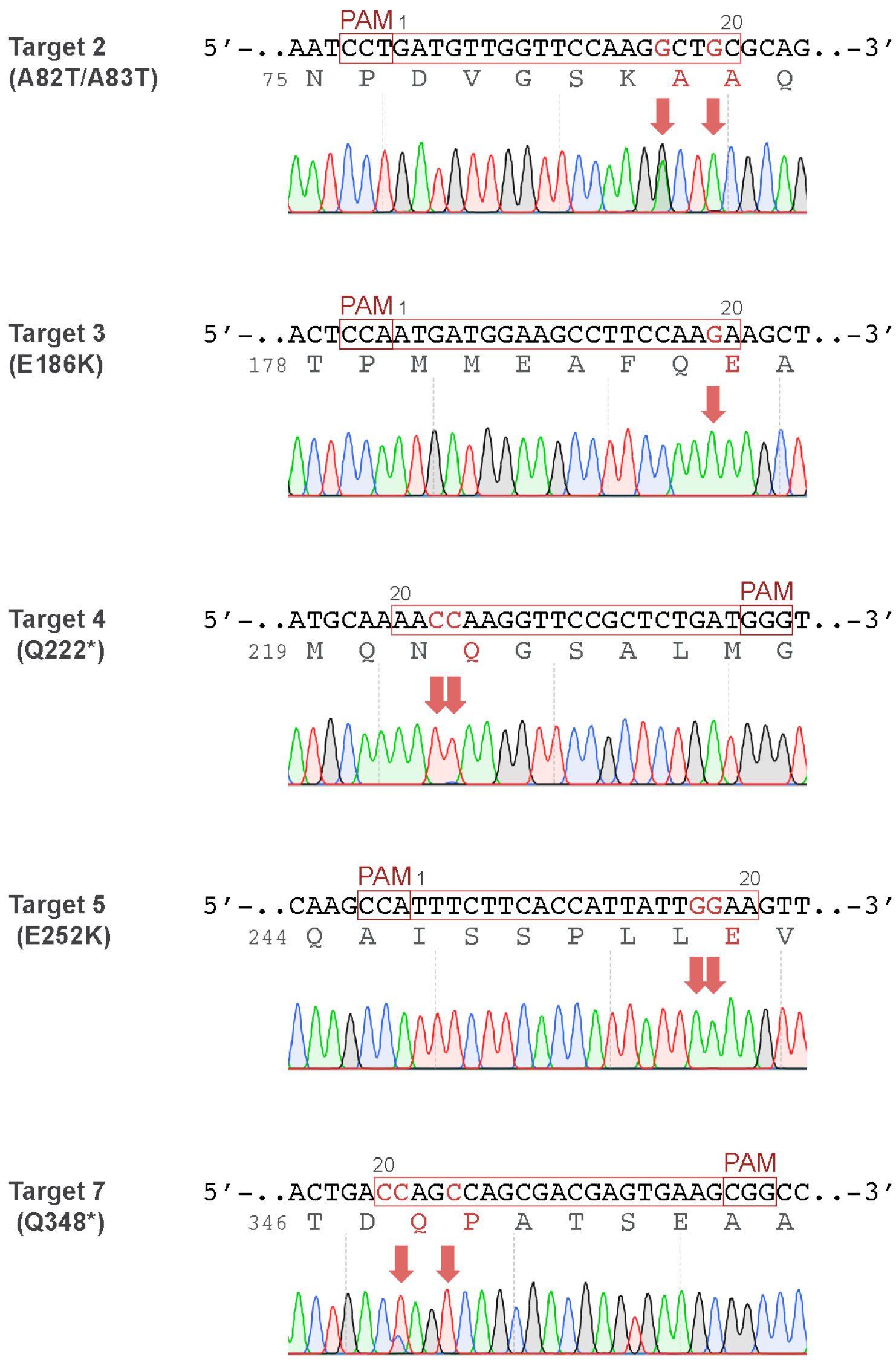

Supplementary Fig. 6

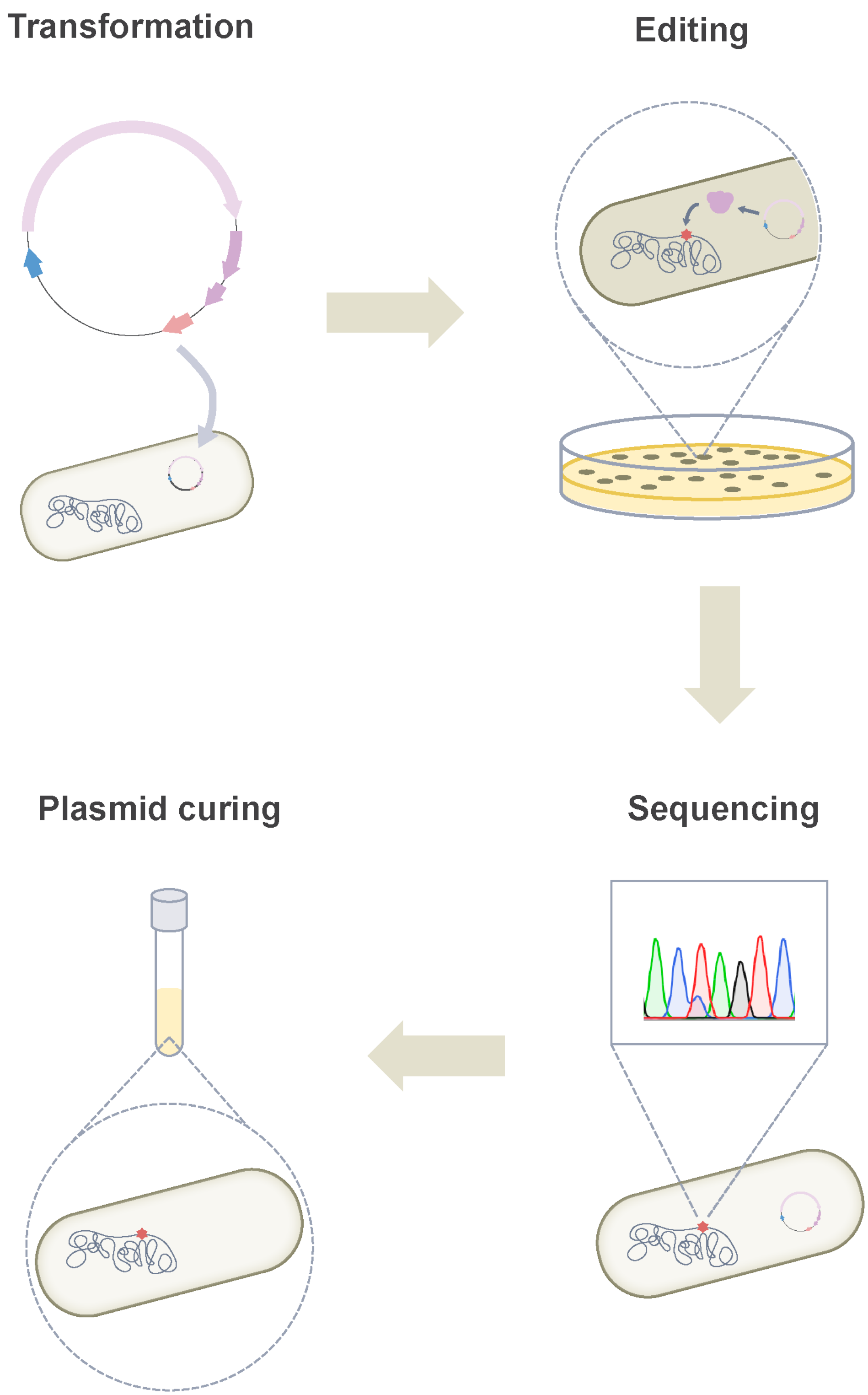

Supplementary Fig. 7

Supplementary Fig. 1

The architecture of a plasmid with the bidirectional promoter and independent sanger sequencing data for the representative plasmids. (a) A bidirectional promoter is located between the nCas9 and tracrRNA genes for driving the effector proteins and tracrRNA in concert. The orientation of tracrRNA with the bidirectional promoter is opposite from the one with a separated promoter. (b) Independent sanger sequencing data of the random 8 colonies of the transformants with the representative plasmids (pYK02 and pYK06) were shown

Supplementary Fig. 2

PAM-flexible Target-AID with NG-Cas9 vector and its self-targeting effect. (a) pYK12 was developed based on pYK6 by replacing coding sequence for Cas9. Sanger sequencing spectra of the constructed plasmids containing targeting sequences (*upp* (top) and *ftsZ* (bottom)) showed mixed populations at the crRNA region. (b) The target sequences for *upp* and *ftsZ* on the genome of *L. plantarum*. (c) The editing frequencies are determined by sequencing eight randomly selected colonies. The highest frequency among the edited base positions in each colony was plotted as its representative value. The mean editing frequencies are shown as bar graphs, with error bars representing the standard deviation.

Supplementary Fig. 3

Multiplex Target-AID base editing in *L. plantarum.* (a) pYK06-s (single), -d (dual), and -t (triple) were constructed based on pYK06 by inserting in tandem each crRNA expression cassette containing a promoter, a crRNA with a target sequence and a terminator. (b) Three target sequences are selected from *urdA* gene and editable cytosines are shown in red. (c) The editing

frequencies at the three sites are determined by sequencing six randomly selected colonies and plotted at each editable base position. The mean editing frequencies (bars) are shown with standard deviation (error bars)

Supplementary Fig. 4

Transformation efficiencies of *Lactobacillus plantarum* and *Lactobacillus gasseri* with plasmids pYN_Lg2-4 and pYK07. Plates show transformations of *L. plantarum* WCFS1 (top) and *L. gasseri* ATCC 33323 (bottom) with pYN_Lg2-4 (Left) and pYK07 (Right). Plates were incubated at 30°C for 3 for *L. plantarum* and 7 days for *L. gasseri*

Supplementary Fig. 5

urdA editing with Target-AID in *L. plantarum*. (a) Sanger sequencing spectra of the four independent colonies edited at *urdA*. (b) The editing frequencies are plotted at each editable base position. The mean editing frequencies (bars) are shown with standard deviation (error bars) from four randomly selected colonies

Supplementary Fig. 6

Sanger sequencing analysis of *ftsZ* targets edited by Target-AID. Sequencing data for the five successfully edited targets are shown, with the wild-type sequences are shown above each chromatogram. The target and PAM sequences are highlighted in boxes. The edited bases are indicated by arrows

Supplementary Fig. 7

Experimental workflow for editing and establishing edited strains in *Lactobacillus*. Target-AID expressing vectors are introduced into cells via electroporation. The transformants with the plasmid are selected on the plate, and randomly selected colonies undergoes sequencing to investigate editing at the target sites. Edited colonies are cultured in antibiotic-free medium to remove the Target-AID plasmid, and plasmid-free strains are confirmed by PCR and sequencing to verify the absence of plasmid and the presence of the desired mutations

Supplementary Note 1

pYN_Lg4

Teal: Pldh(Lc) promoter

Blue: tracrRNA

Green: Ppgm(La) promoter

Lime: P23 (Ll) promoter

Maroon: crRNA direct repeat

Lavender: pIB184-derived seq (*repD*, *repE*, and *ermB*)

Cyan: ColE1 ori

Yellow: KanR

Uppercase: ORF

```
atgatggacggattttgccttttccgtccagagcagtataccatactgacgccttgcgtctgtg
cgagttgaccgtgggaactgttgaatttcccacattcgtgaaaacagatgctggaaacgcttac
gttattggaagcttgcttgaaacaggattcacaagtcttgctgtagtaaggctcgacgccattt
tttgacaatggcaaaatcatgaaaaagtctatcaaatttgtttcagggaattgataatgtgtta
tactcaacgtgaaatgcagtttgcatgcacataagaaaggatgatatcaccgacgtcggaacca
ttcaaaacagcatagcaagtttaaataaggctagtccgttatcaacttgaaaaagtggcaccga
gtcggtgctttttttcgatcgcTGCGACAAGTAATAAACTAAACAAAACAACTACAAAATATTT
CTTTTTGTTTTTCATGATTTTTACACTTCTCTTAGTATGCTTTTGTTATAAGTTAGCACAAAAA
AGCAGAAAATAAAAAGTAGAAATAAAAAAAGATGTTTTTTTGCCCATATCTCTATGAAAAAAAC
TGTGAAATGTGTAAAATATGGATGAAACATTGAATTTAAAAGGAGATATTTCATGTCAAAATTA
GTTTTAATCCGTCACGGTCAAAGTGAATGGAACCTTTCAAACCAATTTACTGGTTGGGTTGACG
TTAACCTTTCAGAAAAAGGTGTTGAAGAAGCTAAGAAGGCTgtcgactatcCGGCCGaagtcct
gcagggaaaagccctgacaacccttgttcctaaaaaggaataagcgttcggtcagtaaataata
gaaataaaaaatcagacctaagactgatgacaaaaagagaaattttgataaaatagtcttaga
attaaattaaaaagggaggccaaatataatgaaaaatatgaatgacaatgatgttagagacccg
ggtctcaGTTTAAGAGCTATGCTGTTTTGAATGGTCCCAAAACGTAAAAAAAACCCatgcatac
tagtttaatcactttgactagcaaatactaacaacaagacacacacaccaaaaatcaaaaattc
actactttagttaaaaaccacgtaaccacaagaactaatccaatccatgtaatcgggttcttc
aaatatttctccaagatttcctcctctaatatgctcaacttaaatgacctattcaataaatct
attatgctgctaaatagtttataggacaaataagtatactctaatgacctataaaagatagaaa
attaaaaaatcaagtgttcgcttcgctctcactgcccctcgacgttttagtagcctttccctca
cttcgttcagtccaagccaactaaaagttttcggctactctctccttctcccctaataatta
attaaaatcttactctgtatatttctgctaatcattcgctaaacagcaaagaaaaacaaacac
gtatcatagatataaatgtaatggcatagtgcgggttttatttTCAGCCTGTATCATAGCTAAA
CAAATCGAGTTGTGTGTCCGTTTTAGGGCGTTCTGCTAGCTTGTTTAAAGTCTCTTGAATGAAT
```

GTATGCTCTAAGTCAAAAGAATTTGTCAGCGCCTTTATATAGCTTTCTTTTTCTTCTTTTTTTA
CTTTAATGATCGATAGCAACAATGATTTAACACTAGCAAGTTGAATGCCACCATTTCTTCCTGG
TTTAATCTTAAAGAAAATTTCCTGATTCGCCTTCAGTACCTTCAGCAATTTATCTAATGTCCGT
TCAGGAATGCCTAGCACTTCTCTAATCTCTTTTTTGGTCGTCACTAAATAAGGCTTGTATACAT
CGCTTTTTTTCGCTAATATAAGCCATTAAATCTTCTTTCCATTCTGACAAATGAACACGTTGACG
TTCGCTTCTTTTTTTCTTGAATTTAAACCACCCTTGACGGACAAATAAATCTTTACTGGTTAAA
TCACTTGATACCCAAGCTTTGCAAAGAATGGTAATGTATTCCCTATTAGCCCCTTGATAGTTTT
CTGAATAGGCACTTCTAACAATTTTGATTACTTCTTTTTCTTCTAAGGGTTGATCTAATCGATT
ATTAAACTCAAACATATTATATTCGCACGTTTCGATTGAATAGCCTGAACTAAAGTAGGCTAAA
GAGAGGGTAAACATGACGTTATTACGCCCTATTAAACCCTTTTCTCCTGAAAATTTCGTTTCGT
GCAATAAGAGATTAAACCAGGGTTCATCTACTTGTTTTTTGCCTTCTGTACCGCTTAAAACCGT
TAGACTTGAACGAGTAAAGCCCTTATTATCTGTTTGTTTGAAAGACCAATCTTGCCATTCTTTG
AAAGAATAACGGTAATTAGGATCAAAAAATTCTACATTGTCCGTTCTTGGTATGCGAGCAATAC
CAAAATGATTACACGTTAGATCAACTGGCAAAGACTTTCCAAAATATTCTCGGATATTTTGCGA
AATTATTTTGGCTGCTTTGACAGATTTAAATTCTGATTTTGAAGTCACATAGACTGGCGTTTCT
AAAACAAAATATGCTTGATAACCTTTATCAGATTTGATAATCATAGTAGGCATAAAACCTAAAT
CAATAGCGGTTGTTAAAATATCGCTTGCTGAAATAGTTTCTTTTGCCGTGTGAATATCAAAATC
AATAAAGAAGGTATTGATTTGTCTTAAATTGTTTTCAGAATGTCCTTTCGTGTATGAACGGTTT
TCGTCTGCATACGTTCCATAACGATAAACGTTGGGTGTCCAATGTGTAAATGTATCTTGATTTT
CTTGAATCGCTTCCTCGGAAGTCAGAACAACACCACGACCGCCAATCATGCTTGATTTTGAGCG
ATACGCAAAAATAGCCCCTTTGCTTTTACCTGGCTTGGTAGTGATTGAGCGAATTTTACTATTT
TTAAATTTGTACTTTAACAAGCCGTCATGAAGCACAGTTTCTACAACAAAAGGGATATTCATTtc
agctgttctcctttcctataaatcctataaaataggttgtTTAATTAACTTGGTTTGCTTTTTC
ATTCAACTGTTTCAATATTGCATGTTTTGAAAAAGATTTTTTTCCTTTATAAGTCAATTTTTTT
CCACTAATCGAATAAATTATTTTGTTATTTTCTATTAACTTATATATATAATCTTCCCCCTCCG
AAGAAAAATACTTATCTGATTTTGTTTCTAAGTAGATATTTCTCTTTTCTAACTCTTTCTTAAA
CGTTTCTAGTGTATAGATATTTGCTAATTTTCTTATCTCcaataaactatttttttatataagtt
ttacattcatcatgattcatacaaactccaccttctataaatgaatacaaaaaaagcaatcaaa
cgatttccgattgattgcttaacaattcttaaattcagtagcttagatacttgaaaactctctg
atttccctatataatgatagtacggttatataccgtcttcaaacaaagttaattaaataacttc
ttacgagggaagagttcatctgactaactgataagcgttggtttggcaatcttatcgggctatg
catttataaaatgtcgtcaaacattttataaatgtgtcatggctctttttttcgtttctattcag
ttcgttgtttcgttatatctagtataccgcttttaaaaaaaataagcaacgatttcgtgcatta
ttcacacgaagtcattgctttttttcttcttccatttctaaatccaatgttacttgttctgattc
tgtttctggttctggttctgttggctcatttgggattaaatccactactagcgttgagttagtt
aactttgcaatttgttctagtgtttttatggttggatctgattttcctgattctattcgtgaat
aatttgatctactcatttctaattcttgggtaccgccagcatttcggaaaaaaaccacgctaa
ggatttttttctataaaaagagccgttatattaagaataaaacggctcttttatacgtaaaggac
gtaaattcatttgcccagtgtcatgtaatccttcaaatttgtattctccaagaaaattgatatg
ttcccatcctaacggccacgcatatggcattaaatcttctctaaattctcctcttgcttttaat
tcttctacggctttttccatatatacagtgttccacacacttatagcgttaataattatgttta
gtgcactagctctttgtaactggtcttggagagcacgttctctaaattctccacgttgtccaaa
aaatataattctagctaatgcattgattgcttctcctttatttaaacctttttgaacccgtctc
cttacggctttattagatatgtaatccagcgtaaagagggttttctcgattcgtcccatttctc
caagtgctgttgcgagtttattttgtcttgcatatgatccgagcttccccatgataagagcgct
agggacctctttagctccttggaagctgtcagtagtatacctaataatttatctacattccctt
tagtaacgtgtaactttccaaatttacaaaagcgactcatagaaTTATTTCCTCCCGTTAAATA

ATAGATAACTATTAAAAATAGACAATACTTGCTCATAAGTAACGGTACTTAAATTGTTTACTTT
GGCGTGTTTCATTGCTTGATGAAACTGATTTTTAGTAAACAGTTGACGATATTCTCGATTGACC
CATTTTGAAACAAAGTACGTATATAGCTTCCAATATTTATCTGGAACATCTGTGGTATGGCGGG
TAAGTTTTATTAAGACACTGTTTACTTTTGGTTTAGGATGAAAGCATTCCGCTGGCAGCTTAAG
CAATTGCTGAATCGAGACTTGAGTGTGCAAGAGCAACCCTAGTGTTCGGTGAATATCCAAGGTA
CGCTTGTAGAATCCTTCTTCAACAATCAGATAGATGTCAGACGCATGGCTTTCAAAAACCACTT
TTTTAATAATTTGTGTGCTTAAATGGTAAGGAATACTCCCAACAATTTTATACCTCTGTTTGTT
AGGGAATTGAAACTGTAGAATATCTTGGTGAATTAAAGTGACACGAGTATTCAGTTTTAATTTT
TCTGACGATAAGTTGAATAGATGACTGTCTAATTCAATAGACGTTACCTGTTTACTTATTTTAG
CCAGTTTCGTCGTTAAATGCCCTTTACCTGTTCCAATTTCGTAAACGGTATCGGTTTCTTTTAA
ATTCAATTGTTTTATTATTTGGTTGAGTACTTTTTCACTCGTTAAAAAGTTTTGAGAATATTTT
ATATTTTTGTTCATgtaatcactccttcttaattacaaatttttagcatctaatttaacttcaa
ttcctattatacaaaattttaagatactgcactatcaacacactcttaagtttgcttctaagtc
ttatttccataacttcttttacgtttccgccattctttgctgtttcgatttttatgatatggtg
caagtcagcacgaacacgaaccgtcttatctcccattatatctttttttgcactgattggtgta
tcatttcgtttttctttttgtgcgcttcttgataaaaggatagtaattcattcctggttgcaa
attttgaaaaccgctacggatcgcatctttttctaaactagggcccacaacggcgctgccgacc
tgggtcagcatcaatttccataggctccgcccccctgacgagcatcacaaaaatcgacgctcaa
gtcagaggtggcgaaacccgacaggactataaagataccaggcgtttccccctggaagctccct
cgtgcgctctcctgttccgaccctgccgcttaccggatacctgtccgcctttctcccttcggga
agcgtggcgctttctcatagctcacgctgtaggtatctcagttcggtgtaggtcgttcgctcca
agctgggctgtgtgcacgaaccccccgttcagcccgaccgctgcgccttatccggtaactatcg
tcttgagtccaacccggtaagacacgacttatcgccactggcagcagccactggtaacaggatt
agcagagcgaggtatgtaggcggtgctacagagttcttgaagtggtggcctaactacggctaca
ctagaagaacagtatttggtatctgcgctctgctgaagccagttaccttcggaaaaagagttgg
tagctcttgatccggcaaacaaaccaccgctggtagcggtggtttttttgtttgcaagcagcag
attacgcgcagaaaaaaaggatctcaaGAAGATCCTTTGATCTTTTCTACGGGGTCTGACGCTC
AGTGGAACGAAAACTCACGTTAAGGGATTTTGGTCATGAGATTATCAAAAAGGATCTTCACCTA
GATCCTTTTAAATTAAAAATGAAGTTTTAAATCAATCTAAAGTATATATGAGTAAACTTGGTCT
GACAGTTAGAAAAACTCATCGAGCATCAAATGAAACTGCAATTTATTCATATCAGGATTATCAA
TACCATATTTTTGAAAAAGCCGTTTCTGTAATGAAGGAGAAAACTCACCGAGGCAGTTCCATAG
GATGGCAAGATCCTGGTATCGGTCTGCGATTCCGACTCGTCCAACATCAATACAACCTATTAAT
TTCCCCTCGTCAAAAATAAGGTTATCAAGTGAGAAATCACCATGAGTGACGACTGAATCCGGTG
AGAATGGCAAAAGTTTATGCATTTCTTTCCAGACTTGTTCAACAGGCCAGCCATTACGCTCGTC
ATCAAAATCACTCGCATCAACCAAACCGTTATTCATTCGTGATTGCGCCTGAGCGAGACGAAAT
ACGCGGTCGCTGTTAAAAGGACAATTACAAACAGGAATCGAATGCAACCGGCGCAGGAACACTG
CCAGCGCATCAACAATATTTTCACCTGAATCAGGATATTCTTCTAATACCTGGAATGCTGTTTT
CCCAGGGATCGCAGTGGTGAGTAACCATGCATCATCAGGAGTACGGATAAAATGCTTGATGGTC
GGAAGAGGCATAAATTCCGTCAGCCAGTTTAGTCTGACCATCTCATCTGTAACATCATTGGCAA
CGCTACCTTTGCCATGTTTCAGAAACAACTCTGGCGCATCGGGCTTCCCATACAATCGATAGAT
TGTCGCACCTGATTGCCCGACATTATCGCGAGCCCATTTATACCCATATAAATCAGCATCCATG
TTGGAATTTAATCGCGGCCTAGAGCAAGACGTTTCCCGTTGAATATGGCTCATactcttccttt
ttcaatattattgaagcatttatcagggttattgtctcatgagcggatacatatttgaatgtat
ttagaaaaataaacaaataggggttccgcgaccggttatcg

Supplementary Note 2

pYK01

Blue: tracrRNA
Green: gyrA-ldh (Sm) bidirectional promoter
Pink: nCas9(D10A)
Brown: SH3
Red: PmCDA1
Apricot: SHL
Purple: UGI
Lime: P23 (Ll) promoter
Maroon: crRNA direct repeat
Lavender: pIB184-derived seq (*repD*, *repE*, and *ermB*)
Cyan: ColE1 ori
Yellow: KanR

Uppercase: ORF
Underline: gRNA spacer sequence (upp)

ggtaaaaaagcaccgactcggtgccactttttcaagttgataacggactagccttatttaaac
ttgctatgctgttttgaatggttcttttatattatatcacaaataaggctctttttcagctatt
ctactatagttttccgctgagaaaggtaaatattagtgactttcttaacaaaaagtgttagaat
gaaaatgtatagaatatatacttaataaattataagtcgaccggcccattttaggaggcaaaaA
TGGATAAGAAATACTCAATAGGCTTAGCTATCGGCACAAATAGCGTCGGATGGGCGGTGATCAC
TGATGAATATAAGGTTCCGTCTAAAAAGTTCAAGGTTCTGGGAAATACAGACCGCCACAGTATC
AAAAAAAATCTTATAGGGGCTCTTTTATTTGACAGTGGAGAGACAGCGGAAGCGACTCGTCTCA
AACGGACAGCTCGTAGAAGGTATACACGTCGGAAGAATCGTATTTGTTATCTACAGGAGATTTT
TTCAAATGAGATGGCGAAAGTAGATGATAGTTTCTTTCATCGACTTGAAGAGTCTTTTTTGGTG
GAAGAAGACAAGAAGCATGAACGTCATCCTATTTTTGGAAATATAGTAGATGAAGTTGCTTATC
ATGAGAAATATCCAACTATCTATCATCTGCGAAAAAAATTGGTAGATTCTACTGATAAAGCGGA

TTTGCGCTTAATCTATTTGGCCTTAGCGCATATGATTAAGTTTCGTGGTCATTTTTTGATTGAG
GGAGATTTAAATCCTGATAATAGTGATGTGGACAAACTATTTATCCAGTTGGTACAAACCTACA
ATCAATTATTTGAAGAAAACCCTATTAACGCAAGTGGAGTAGATGCTAAAGCGATTCTTTCTGC
ACGATTGAGTAAATCAAGACGATTAGAAAATCTCATTGCTCAGCTCCCCGGTGAGAAGAAAAAT
GGCTTATTTGGGAATCTCATTGCTTTGTCATTGGGTTTGACCCCTAATTTTAAATCAAATTTTG
ATTTGGCAGAAGATGCTAAATTACAGCTTTCAAAAGATACTTACGATGATGATTTAGATAATTT
ATTGGCGCAAATTGGAGATCAATATGCTGATTTGTTTTTGGCAGCTAAGAATTTATCAGATGCT
ATTTTACTTTCAGATATCCTAAGAGTAAATACTGAAATAACTAAGGCTCCCCTATCAGCTTCAA
TGATTAAACGCTACGATGAACATCATCAAGACTTGACTCTTTTAAAAGCTTTAGTTCGACAACA
ACTTCCAGAAAAGTATAAAGAAATCTTTTTTGATCAATCAAAAAACGGATATGCAGGTTATATT
GATGGGGGAGCTAGCCAAGAAGAATTTTATAAATTTATCAAACCAATTTTAGAAAAAATGGATG
GTACTGAGGAATTATTGGTGAAACTAAATCGTGAAGATTTGCTGCGCAAGCAACGGACCTTTGA
CAACGGCTCTATTCCCCATCAAATTCACTTGGGTGAGCTGCATGCTATTTTGAGAAGACAAGAA
GACTTTTATCCATTTTTAAAAGACAATCGTGAGAAGATTGAAAAAATCTTGACTTTTCGAATTC
CTTATTATGTTGGTCCATTGGCGCGTGGCAATAGTCGTTTTGCATGGATGACTCGGAAGTCTGA
AGAAACAATTACCCCATGGAATTTTGAAGAAGTTGTCGATAAAGGTGCTTCAGCTCAATCATTT
ATTGAACGCATGACAAACTTTGATAAAAATCTTCCAAATGAAAAAGTACTACCAAAACATAGTT
TGCTTTATGAGTATTTTACGGTTTATAACGAATTGACAAAGGTCAAATATGTTACTGAAGGAAT
GCGAAAACCAGCATTTCTTTCAGGTGAACAGAAGAAAGCCATTGTTGATTTACTCTTCAAAACA
AATCGAAAAGTAACCGTTAAGCAATTAAAAGAAGATTATTTCAAAAAAATAGAATGTTTTGATA
GTGTTGAAATTTCAGGAGTTGAAGATAGATTTAATGCTTCATTAGGTACCTACCATGATTTGCT
AAAAATTATTAAAGATAAAGATTTTTTGGATAATGAAGAAAATGAAGATATCTTAGAGGATATT
GTTTTAACATTGACCTTATTTGAAGATAGGGAGATGATTGAGGAAAGACTTAAAACATATGCTC
ACCTCTTTGATGATAAGGTGATGAAACAGCTTAAACGTCGCCGTTATACTGGTTGGGGACGTTT
GTCTCGAAAATTGATTAATGGTATTAGGGATAAGCAATCTGGCAAAACAATATTAGATTTTTTG
AAATCAGATGGTTTTGCCAATCGCAATTTTATGCAGCTGATCCATGATGATAGTTTGACATTTA
AAGAAGACATTCAAAAAGCACAAGTGTCTGGACAAGGCGATAGTTTACATGAACATATTGCAAA
TTTAGCTGGTAGCCCTGCTATTAAAAAAGGTATTTTACAGACTGTAAAAGTTGTTGATGAATTG
GTCAAAGTAATGGGGCGGCATAAGCCAGAAAATATCGTTATTGAAATGGCACGTGAAAATCAGA
CAACTCAAAAGGGCCAGAAAAATTCGCGAGAGCGTATGAAACGAATCGAAGAAGGTATCAAAGA
ATTAGGAAGTCAGATTCTTAAAGAGCATCCTGTTGAAAATACTCAATTGCAAAATGAAAAGCTC
TATCTCTATTATCTCCAAAATGGAAGAGACATGTATGTGGACCAAGAATTAGATATTAATCGTT

TAAGTGATTATGATGTCGATcaCATTGTTCCACAAAGTTTCCTTAAAGACGATTCAATAGACAA
TAAGGTCTTAACGCGTTCTGATAAAAATCGTGGTAAATCGGATAACGTTCCAAGTGAAGAAGTA
GTCAAAAAGATGAAAAACTATTGGAGACAACTTCTAAACGCCAAGTTAATCACTCAACGTAAGT
TTGATAATTTAACGAAAGCTGAACGTGGAGGTTTGAGTGAACTTGATAAAGCTGGTTTTATCAA
ACGCCAATTGGTTGAAACTCGCCAAATCACTAAGCATGTGGCACAAATTTTGGATAGTCGCATG
AATACTAAATACGATGAAAATGATAAACTTATTCGAGAGGTTAAAGTGATTACCTTAAAATCTA
AATTAGTTTCTGACTTCCGAAAAGATTTCCAATTCTATAAAGTACGTGAGATTAACAATTACCA
TCATGCCCATGATGCGTATCTAAATGCCGTCGTTGGAACTGCTTTGATTAAGAAATATCCAAAA
CTTGAATCGGAGTTTGTCTATGGTGATTATAAAGTTTATGATGTTCGTAAAATGATTGCTAAGT
CTGAGCAAGAAATAGGCAAAGCAACCGCAAAATATTTCTTTTACTCTAATATCATGAACTTCTT
CAAAACAGAAATTACACTTGCAAATGGAGAGATTCGCAAACGCCCTCTAATCGAAACTAATGGG
GAAACTGGAGAAATTGTCTGGGATAAAGGGCGAGATTTTGCCACAGTGCGCAAAGTATTGTCCA
TGCCCCAAGTCAATATTGTCAAGAAAACAGAAGTACAGACAGGCGGATTCTCCAAGGAGTCAAT
TTTACCAAAAAGAAATTCGGACAAGCTTATTGCTCGTAAAAAAGACTGGGATCCAAAAAAATAT
GGTGGTTTTGATAGTCCAACGGTAGCTTATTCAGTCCTAGTGGTTGCTAAGGTGGAAAAAGGGA
AATCGAAGAAGTTAAAATCCGTTAAAGAGTTACTAGGGATCACAATTATGGAAAGAAGTTCCTT
TGAAAAAAATCCGATTGACTTTTTAGAAGCTAAAGGATATAAGGAAGTTAAAAAAGACTTAATC
ATTAAACTACCTAAATATAGTCTTTTTGAGTTAGAAAACGGTCGTAAACGGATGCTGGCTAGTG
CCGGAGAATTACAAAAAGGAAATGAGCTGGCTCTGCCAAGCAAATATGTGAATTTTTTATATTT
AGCTAGTCATTATGAAAAGTTGAAGGGTAGTCCAGAAGATAACGAACAAAAACAATTGTTTGTG
GAGCAGCATAAGCATTATTTAGATGAGATTATTGAGCAAATCAGTGAATTTTCTAAGCGTGTTA
TTTTAGCAGATGCCAATTTAGATAAAGTTCTTAGTGCATATAACAAACATAGAGACAAACCAAT
ACGTGAACAAGCAGAAAATATTATTCATTTATTTACGTTGACGAATCTTGGAGCTCCCGCTGCT
TTTAAATATTTTGATACAACAATTGATCGTAAACGATATACGTCTACAAAAGAAGTTTTAGATG
CCACTCTTATCCATCAATCCATCACTGGTCTTTATGAAACACGCATTGATTTGAGTCAGCTAGG
AGGTGACGGTGGAGGAGGTTCTGGAGGTGGAGGTTCTGCTGAGTATGTGCGAGCCCTCTTTGAC
TTTAATGGGAATGATGAAGAGGATCTTCCCTTTAAGAAAGGAGACATCCTGAGAATCCGGGATA
AGCCTGAGGAGCAGTGGTGGAATGCAGAGGACAGCGAAGGAAAGAGGGGGATGATTCCTGTCCC
TTACGTGGAGAAGTATTCCGGAGACTATAAGGACCACGACGGAGACTACAAGGATCATGATATT
GATTACAAAGACGATGACGATAAGTCTAGGCTCGAGTAGgaggtctagaATGACCGACGCTGAG
TACGTGAGAATCCATGAGAAGTTGGACATCTACACGTTTAAGAAACAGTTTTTCAACAACAAAA
AATCCGTGTCGCATAGATGCTACGTTCTCTTTGAATTAAAACGACGGGGTGAACGTAGAGCGTG

TTTTTGGGGCTATGCTGTGAATAAACCACAGAGCGGGACAGAACGTGGCATTCACGCCGAAATCTTTAGCATTAGAAAAGTCGAAGAATACCTGCGCGACAACCCCGGACAATTCACGATAAATTGGTACTCATCCTGGAGTCCTTGTGCAGATTGCGCTGAAAAGATCTTAGAATGGTATAACCAGGAGCTGCGGGGGAACGGCCACACTTTGAAAATCTGGGCTTGCAAACTCTATTACGAGAAAAATGCGAGGAATCAAATTGGGCTGTGGAACCTCAGAGATAACGGGGTTGGGTTGAATGTAATGGTAAGTGAACACTACCAATGTTGCAGGAAAATATTCATCCAATCGTCGCACAATCAATTGAATGAGAATAGATGGCTTGAGAAGACTTTGAAGCGAGCTGAAAAACGACGGAGCGAGTTGTCCATTATGATTCAGGTAAAAATACTCCACACCACTAAGAGTCCTGCTGTTGGCGGTGGTGGTAGTCCACCTCCAGCACTGCCTCCAAAGAGGAGGAGATGAatATGACCAACCTTTCCGACATCATAGAGAAGGAAACAGGCAAACAGTTGGTCATCCAAGAGTCGATACTCATGCTTCCTGAAGAAGTTGAGGAGGTCATTGGGAATAAGCCGGAAAGTGACATTCTCGTACACACTGCGTATGATGAGAGCACCGATGAGAACGTGATGCTGCTCACGTCAGATGCCCCAGAGTACAAACCCTGGGCTCTGGTGATTCAGGACTCTAATGGAGAGAACAAGATCAAGATGCTAGGACTGGTTGCATAAcggccgaagtcctgcaggcttgttcctaaaaaggaataagcgttcggtcagtaaataatagaaataaaaaatcagacctaagactgatgacaaaaagagaaaattttgataaaatagtcttttcaacataaattgacgatcattgtttaagagctatgctgttttgaatggtcccaaaacgtaaaaaaacccatgcatactagtttaatcactttgactagcaaatactaacaacaagacacacacaccaaaaatcaaaaattcactactttagttaaaaaccacgtaaccacaagaactaatccaatccatgtaatcgggttcttcaaatatttctccagattttcctcctctaatatgctcaacttaaatgacctattcaataaatctattatgctgctaaatagtttataggacaaataagtatactctaatgacctataaaagatagaaaattaaaaaatcaagtgttcgcttcgctctcactgcccctcgacgttttagtagcctttccctcacttcgttcagtccaagccaactaaaagttttcgggctactctctccttctcccccctaataattaattaaaatcttactctgtatatttctgctaatcattcgctaaacagcaaagaaaaaacaaacacgtatcatagatataaatgtaatggcatagtgcgggttttatttTCAGCCTGTATCATAGCTAAACAAATCGAGTTGTGTGTCCGTTTTAGGGCGTTCTGCTAGCTTGTTTAAAGTCTCTTGAATGAATGTATGCTCTAAGTCAAAAGAATTTGTCAGCGCCTTTATATAGCTTTCTTTTTCTTCTTTTTTTACTTTAATGATCGATAGCAACAATGATTTAACACTAGCAAGTTGAATGCCACCATTTCTTCCTGGTTTAATCTTAAAGAAAATTTCCTGATTCGCCTTCAGTACCTTCAGCAATTTATCTAATGTCCGTTCAGGAATGCCTAGCACTTCTCTAATCTCTTTTTTGGTCGTCACTAAATAAGGCTTGTATACATCGCTTTTTTCGCTAATATAAGCCATTAAATCTTCTTTCCATTCTGACAAATGAACACGTTGACGTTCGCTTCTTTTTTTTCTTGAATTTAAACCACCCTTGACGGACAAATAAATCTTTACTGGTTAAATCACTTGATACCCAAGCTTTGCAAAGAATGGTAATGTATTCCCTATTAGCCCCTTGATAGTTTTCTGAATAGGCACTTCTAACAAT

TTTGATTACTTCTTTTTCTTCTAAGGGTTGATCTAATCGATTATTAAACTCAAACATATTATAT
TCGCACGTTTCGATTGAATAGCCTGAACTAAAGTAGGCTAAAGAGAGGGTAAACATGACGTTAT
TACGCCCTATTAAACCCTTTTCTCCTGAAAATTTCGTTTCGTGCAATAAGAGATTAAACCAGGG
TTCATCTACTTGTTTTTTGCCTTCTGTACCGCTTAAAACCGTTAGACTTGAACGAGTAAAGCCC
TTATTATCTGTTTGTTTGAAAGACCAATCTTGCCATTCTTTGAAAGAATAACGGTAATTAGGAT
CAAAAAATTCTACATTGTCCGTTCTTGGTATGCGAGCAATACCAAAATGATTACACGTTAGATC
AACTGGCAAAGACTTTCCAAAATATTCTCGGATATTTTGCGAAATTATTTTGGCTGCTTTGACA
GATTTAAATTCTGATTTTGAAGTCACATAGACTGGCGTTTCTAAAACAAAATATGCTTGATAAC
CTTTATCAGATTTGATAATCATAGTAGGCATAAAACCTAAATCAATAGCGGTTGTTAAAATATC
GCTTGCTGAAATAGTTTCTTTTGCCGTGTGAATATCAAAATCAATAAAGAAGGTATTGATTTGT
CTTAAATTGTTTTCAGAATGTCCTTTCGTGTATGAACGGTTTTCGTCTGCATACGTTCCATAAC
GATAAACGTTGGGTGTCCAATGTGTAAATGTATCTTGATTTTCTTGAATCGCTTCCTCGGAAGT
CAGAACAACACCACGACCGCCAATCATGCTTGATTTTGAGCGATACGCAAAAATAGCCCCTTTG
CTTTTACCTGGCTTGGTAGTGATTGAGCGAATTTTACTATTTTTAAATTTGTACTTTAACAAGC
CGTCATGAAGCACAGTTTCTACAACAAAAGGGATATTCATtcagctgttctcctttcctataaa
tcctataaaataggttgtTTAATTAACTTGGTTTGCTTTTTCATTCAACTGTTTCAATATTGCA
TGTTTTGAAAAAGATTTTTTTCCTTTATAAGTCAATTTTTTTCCACTAATCGAATAAATTATTT
TGTTATTTTCTATTAACTTATATATATAATCTTCCCCCTCCGAAGAAAAATACTTATCTGATTT
TGTTTCTAAGTAGATATTTCTCTTTTCTAACTCTTTCTTAAACGTTTCTAGTGTATAGATATTT
GCTAATTTTCTTATCTCcaataaactatttttttatataagttttacattcatcatgattcatac
aaactccaccttctataaatgaatacaaaaaaagcaatcaaacgatttccgattgattgcttaa
caattcttaaattcagtagcttagatacttgaaaactctctgatttccctatataatgatagta
cggttatataccgtcttcaaacaaagttaattaaataacttcttacgagggaagagttcatctg
actaactgataagcgttggtttggcaatcttatcgggctatgcatttataaaatgtcgtcaaac
attttataaatgtgtcatggctctttttttcgtttctattcagttcgttgtttcgttatatctag
tataccgcttttaaaaaaaataagcaacgatttcgtgcattattcacacgaagtcattgctttt
ttcttcttccatttctaaatccaatgttacttgttctgattctgtttctggttctggttctgtt
ggctcatttggattaaatccactactagcgttgagttagttaactttgcaatttgttctagtg
tttttatggttggatctgattttcctgattctattcgtgaataatttgatctactcatttctaa
ttcttggggtaccgccagcatttcggaaaaaaaccacgctaaggatttttttctataaaaagagc
cgttatattaagaataaaacggctcttttatacgtaaaggacgtaaattcatttgcccagtgtc
atgtaatccttcaaatttgtattctccaagaaaattgatatgttcccatcctaacggccacgca

tatggcattaaatcttctctaaattctcctcttgcttttaattcttctacggctttttccatat
atacagtgttccacacacttatagcgttaataattatgtttagtgcactagctctttgtaactg
gtcttggagagcacgttctctaaattctccacgttgtccaaaaaatataattctagctaatgca
ttgattgcttctcctttatttaaaccttttttgaacccgtctccttacggctttattagatatgt
aatccagcgtaaagagggttttctcgattcgtcccatttctccaagtgctgttgcgagtttatt
ttgtcttgcatatgatccgagcttccccatgataagagcgctagggacctctttagctccttgg
aagctgtcagtagtatacctaataatttatctacattccctttagtaacgtgtaactttccaaa
tttacaaaagcgactcatagaaTTATTTCCTCCCGTTAAATAATAGATAACTATTAAAAATAGA
CAATACTTGCTCATAAGTAACGGTACTTAAATTGTTTACTTTGGCGTGTTTCATTGCTTGATGA
AACTGATTTTTAGTAAACAGTTGACGATATTCTCGATTGACCCATTTTGAAACAAAGTACGTAT
ATAGCTTCCAATATTTATCTGGAACATCTGTGGTATGGCGGGTAAGTTTTATTAAGACACTGTT
TACTTTTGGTTTAGGATGAAAGCATTCCGCTGGCAGCTTAAGCAATTGCTGAATCGAGACTTGA
GTGTGCAAGAGCAACCCTAGTGTTCGGTGAATATCCAAGGTACGCTTGTAGAATCCTTCTTCAA
CAATCAGATAGATGTCAGACGCATGGCTTTCAAAAACCACTTTTTTAATAATTTGTGTGCTTAA
ATGGTAAGGAATACTCCCAACAATTTTATACCTCTGTTTGTTAGGGAATTGAAACTGTAGAATA
TCTTGGTGAATTAAAGTGACACGAGTATTCAGTTTTAATTTTTCTGACGATAAGTTGAATAGAT
GACTGTCTAATTCAATAGACGTTACCTGTTTACTTATTTTAGCCAGTTTCGTCGTTAAATGCCC
TTTACCTGTTCCAATTTCGTAAACGGTATCGGTTTCTTTTAAATTCAATTGTTTTATTATTTGG
TTGAGTACTTTTTCACTCGTTAAAAAGTTTTGAGAATATTTTATATTTTTGTTCATgtaatcac
tccttcttaattacaaatttttagcatctaatttaacttcaattcctattatacaaattttaa
gatactgcactatcaacacactcttaagtttgcttctaagtcttatttccataacttcttttac
gtttccgccattctttgctgtttcgatttttatgatatggtgcaagtcagcacgaacacgaacc
gtcttatctcccattatatctttttttgcactgattggtgtatcatttcgtttttctttttgtg
cgcttcttgataaaaggatagtaattcattcctggttgcaaattttgaaaaccgctacggatc
gcatctttttctaaactagggcccacaacggcgctgccgacctgggtcagcatcaatttccata
ggctccgcccccctgacgagcatcacaaaaatcgacgctcaagtcagaggtggcgaaacccgac
aggactataaagataccaggcgtttccccctggaagctccctcgtgcgctctcctgttccgacc
ctgccgcttaccggatacctgtccgcctttctcccttcgggaagcgtggcgctttctcatagct
cacgctgtaggtatctcagttcggtgtaggtcgttcgctccaagctgggctgtgtgcacgaacc
ccccgttcagcccgaccgctgcgccttatccggtaactatcgtcttgagtccaacccggtaaga
cacgacttatcgccactggcagcagccactggtaacaggattagcagagcgaggtatgtaggcg
gtgctacagagttcttgaagtggtggcctaactacggctacactagaagaacagtatttggtat

ctgcgctctgctgaagccagttaccttcggaaaaagagttggtagctcttgatccggcaaacaaaccaccgctggtagcggtggtttttttgtttgcaagcagcagattacgcgcagaaaaaaaggatctcaagaagatcctttgatcttttctacggggtctgacgctcagtggaacgaaaactcacgttaagggattttggtcatgagattatcaaaaaggatcttcacctagatccttttaaattaaaaatgaagttttaaatcaatctaaagtatatatgagtaaacttggtctgacagTTAGAAAAACTCATCGAGCATCAAATGAAACTGCAATTTATTCATATCAGGATTATCAATACCATATTTTTGAAAAAGCCGTTTCTGTAATGAAGGAGAAAACTCACCGAGGCAGTTCCATAGGATGGCAAGATCCTGGTATCGGTCTGCGATTCCGACTCGTCCAACATCAATACAACCTATTAATTTCCCCTCGTCAAAAATAAGGTTATCAAGTGAGAAATCACCATGAGTGACGACTGAATCCGGTGAGAATGGCAAAAGTTTATGCATTTCTTTCCAGACTTGTTCAACAGGCCAGCCATTACGCTCGTCATCAAAATCACTCGCATCAACCAAACCGTTATTCATTCGTGATTGCGCCTGAGCGAGACGAAATACGCGgTCGCTGTTAAAAGGACAATTACAAACAGGAATCGAATGCAACCGGCGCAGGAACACTGCCAGCGCATCAACAATATTTTCACCTGAATCAGGATATTCTTCTAATACCTGGAATGCTGTTTTCCCAGGGATCGCAGTGGTGAGTAACCATGCATCATCAGGAGTACGGATAAAATGCTTGATGGTCGGAAGAGGCATAAATTCCGTCAGCCAGTTTAGTCTGACCATCTCATCTGTAACATCATTGGCAACGCTACCTTTGCCATGTTTCAGAAACAACTCTGGCGCATCGGGCTTCCCATACAATCGATAGATTGTCGCACCTGATTGCCCGACATTATCGCGAGCCCATTTATACCCATATAAATCAGCATCCATGTTGGAATTTAATCGCGGCCTAGAGCAAGACGTTTCCCGTTGAATATGGCTCATactcttcctttttcaatattattgaagcatttatcagggttattgtctcatgagcggatacatatttgaatgtatttagaaaaataaacaaatagggGTTCCGCGACC

Supplementary Note 3

pYK06

Teal: Pldh(Lc) promoter

Blue: tracrRNA

Green: Ppgm(La) promoter

Pink: nCas9(D10A)

Brown: SH3

Red: PmCDA1

Apricot: SHL

Purple: UGI

Lime: rrn4a(Lp) promoter

Maroon: crRNA direct repeat

Magenta: rrnB(Ec) terminator

Lavender: pIB184-derived seq (*repD*, *repE*, and *ermB*)

Cyan: ColE1 ori

Yellow: KanR

Uppercase: ORF

Underline: gRNA spacer sequence (upp)

```
atgatggacggattttgccttttccgtccagagcagtataccatactgacgccttgcgtctgtg
cgagttgaccgtgggaactgttgaatttcccacattcgtgaaaacagatgctggaaacgcttac
gttattggaagcttgcttgaaacaggattcacaagtcttgctgtagtaaggctcgacgccattt
tttgacaatggcaaaatcatgaaaaagtctatcaaatttgtttcagggaattgataatgtgtta
tactcaacgtgaaatgcagtttgcatgcacataagaaaggatgatatcaccgacgtcggaacca
ttcaaaacagcatagcaagtttaaataaggctagtccgttatcaacttgaaaaagtggcaccga
gtcggtgctttttttcgatcgcgttagcacaaaaagcagaaaataaaaagtagaaataaaaaa
agatgtttttttgcccatatctctatgaaaaaaactgtgaaatgtgtaaaatatggatgaaaca
ttgaatttaaaaggagagtcgaccggcccattttaggaggcaaaaATGGATAAGAAATACTCAA
TAGGCTTAGCTATCGGCACAAATAGCGTCGGATGGGCGGTGATCACTGATGAATATAAGGTTCC
GTCTAAAAAGTTCAAGGTTCTGGGAAATACAGACCGCCACAGTATCAAAAAAAATCTTATAGGG
GCTCTTTTATTTGACAGTGGAGAGACAGCGGAAGCGACTCGTCTCAAACGGACAGCTCGTAGAA
GGTATACACGTCGGAAGAATCGTATTTGTTATCTACAGGAGATTTTTTCAAATGAGATGGCGAA
AGTAGATGATAGTTTCTTTCATCGACTTGAAGAGTCTTTTTTGGTGGAAGAAGACAAGAAGCAT
```

GAACGTCATCCTATTTTTGGAAATATAGTAGATGAAGTTGCTTATCATGAGAAATATCCAACTA
TCTATCATCTGCGAAAAAAATTGGTAGATTCTACTGATAAAGCGGATTTGCGCTTAATCTATTT
GGCCTTAGCGCATATGATTAAGTTTCGTGGTCATTTTTTGATTGAGGGAGATTTAAATCCTGAT
AATAGTGATGTGGACAAACTATTTATCCAGTTGGTACAAACCTACAATCAATTATTTGAAGAAA
ACCCTATTAACGCAAGTGGAGTAGATGCTAAAGCGATTCTTTCTGCACGATTGAGTAAATCAAG
ACGATTAGAAAATCTCATTGCTCAGCTCCCCGGTGAGAAGAAAAATGGCTTATTTGGGAATCTC
ATTGCTTTGTCATTGGGTTTGACCCCTAATTTTAAATCAAATTTTGATTTGGCAGAAGATGCTA
AATTACAGCTTTCAAAAGATACTTACGATGATGATTTAGATAATTTATTGGCGCAAATTGGAGA
TCAATATGCTGATTTGTTTTTGGCAGCTAAGAATTTATCAGATGCTATTTTACTTTCAGATATC
CTAAGAGTAAATACTGAAATAACTAAGGCTCCCCTATCAGCTTCAATGATTAAACGCTACGATG
AACATCATCAAGACTTGACTCTTTTAAAAGCTTTAGTTCGACAACAACTTCCAGAAAAGTATAA
AGAAATCTTTTTTGATCAATCAAAAAACGGATATGCAGGTTATATTGATGGGGGAGCTAGCCAA
GAAGAATTTTATAAATTTATCAAACCAATTTTAGAAAAAATGGATGGTACTGAGGAATTATTGG
TGAAACTAAATCGTGAAGATTTGCTGCGCAAGCAACGGACCTTTGACAACGGCTCTATTCCCCA
TCAAATTCACTTGGGTGAGCTGCATGCTATTTTGAGAAGACAAGAAGACTTTTATCCATTTTTA
AAAGACAATCGTGAGAAGATTGAAAAAATCTTGACTTTTCGAATTCCTTATTATGTTGGTCCAT
TGGCGCGTGGCAATAGTCGTTTTGCATGGATGACTCGGAAGTCTGAAGAAACAATTACCCCATG
GAATTTTGAAGAAGTTGTCGATAAAGGTGCTTCAGCTCAATCATTTATTGAACGCATGACAAAC
TTTGATAAAAATCTTCCAAATGAAAAAGTACTACCAAAACATAGTTTGCTTTATGAGTATTTTA
CGGTTTATAACGAATTGACAAAGGTCAAATATGTTACTGAAGGAATGCGAAAACCAGCATTTCT
TTCAGGTGAACAGAAGAAAGCCATTGTTGATTTACTCTTCAAAACAAATCGAAAAGTAACCGTT
AAGCAATTAAAAGAAGATTATTTCAAAAAAATAGAATGTTTTGATAGTGTTGAAATTTCAGGAG
TTGAAGATAGATTTAATGCTTCATTAGGTACCTACCATGATTTGCTAAAAATTATTAAAGATAA
AGATTTTTTGGATAATGAAGAAAATGAAGATATCTTAGAGGATATTGTTTTAACATTGACCTTA
TTTGAAGATAGGGAGATGATTGAGGAAAGACTTAAAACATATGCTCACCTCTTTGATGATAAGG
TGATGAAACAGCTTAAACGTCGCCGTTATACTGGTTGGGGACGTTTGTCTCGAAAATTGATTAA
TGGTATTAGGGATAAGCAATCTGGCAAAACAATATTAGATTTTTTGAAATCAGATGGTTTTGCC
AATCGCAATTTTATGCAGCTGATCCATGATGATAGTTTGACATTTAAAGAAGACATTCAAAAAG
CACAAGTGTCTGGACAAGGCGATAGTTTACATGAACATATTGCAAATTTAGCTGGTAGCCCTGC
TATTAAAAAAGGTATTTTACAGACTGTAAAAGTTGTTGATGAATTGGTCAAAGTAATGGGGCGG
CATAAGCCAGAAAATATCGTTATTGAAATGGCACGTGAAAATCAGACAACTCAAAAGGGCCAGA
AAAATTCGCGAGAGCGTATGAAACGAATCGAAGAAGGTATCAAAGAATTAGGAAGTCAGATTCT
TAAAGAGCATCCTGTTGAAAATACTCAATTGCAAAATGAAAAGCTCTATCTCTATTATCTCCAA
AATGGAAGAGACATGTATGTGGACCAAGAATTAGATATTAATCGTTTAAGTGATTATGATGTCG
ATcaCATTGTTCCACAAAGTTTCCTTAAAGACGATTCAATAGACAATAAGGTCTTAACGCGTTC
TGATAAAAATCGTGGTAAATCGGATAACGTTCCAAGTGAAGAAGTAGTCAAAAAGATGAAAAAC
TATTGGAGACAACTTCTAAACGCCAAGTTAATCACTCAACGTAAGTTTGATAATTTAACGAAAG
CTGAACGTGGAGGTTTGAGTGAACTTGATAAAGCTGGTTTTATCAAACGCCAATTGGTTGAAAC
TCGCCAAATCACTAAGCATGTGGCACAAATTTTGGATAGTCGCATGAATACTAAATACGATGAA
AATGATAAACTTATTCGAGAGGTTAAAGTGATTACCTTAAAATCTAAATTAGTTTCTGACTTCC
GAAAAGATTTCCAATTCTATAAAGTACGTGAGATTAACAATTACCATCATGCCCATGATGCGTA
TCTAAATGCCGTCGTTGGAACTGCTTTGATTAAGAAATATCCAAAACTTGAATCGGAGTTTGTC
TATGGTGATTATAAAGTTTATGATGTTCGTAAAATGATTGCTAAGTCTGAGCAAGAAATAGGCA
AAGCAACCGCAAAATATTTCTTTTACTCTAATATCATGAACTTCTTCAAAACAGAAATTACACT
TGCAAATGGAGAGATTCGCAAACGCCCTCTAATCGAAACTAATGGGGAAACTGGAGAAATTGTC
TGGGATAAAGGGCGAGATTTTGCCACAGTGCGCAAAGTATTGTCCATGCCCCAAGTCAATATTG
TCAAGAAAACAGAAGTACAGACAGGCGGATTCTCCAAGGAGTCAATTTTACCAAAAAGAAATTC

GGACAAGCTTATTGCTCGTAAAAAAGACTGGGATCCAAAAAAATATGGTGGTTTTGATAGTCCA
ACGGTAGCTTATTCAGTCCTAGTGGTTGCTAAGGTGGAAAAAGGGAAATCGAAGAAGTTAAAAT
CCGTTAAAGAGTTACTAGGGATCACAATTATGGAAAGAAGTTCCTTTGAAAAAAATCCGATTGA
CTTTTTAGAAGCTAAAGGATATAAGGAAGTTAAAAAAGACTTAATCATTAAACTACCTAAATAT
AGTCTTTTTGAGTTAGAAAACGGTCGTAAACGGATGCTGGCTAGTGCCGGAGAATTACAAAAAG
GAAATGAGCTGGCTCTGCCAAGCAAATATGTGAATTTTTTATATTTAGCTAGTCATTATGAAAA
GTTGAAGGGTAGTCCAGAAGATAACGAACAAAAACAATTGTTTGTGGAGCAGCATAAGCATTAT
TTAGATGAGATTATTGAGCAAATCAGTGAATTTTCTAAGCGTGTTATTTTAGCAGATGCCAATT
TAGATAAAGTTCTTAGTGCATATAACAAACATAGAGACAAACCAATACGTGAACAAGCAGAAAA
TATTATTCATTTATTTACGTTGACGAATCTTGGAGCTCCCGCTGCTTTTAAATATTTTGATACA
ACAATTGATCGTAAACGATATACGTCTACAAAAGAAGTTTTAGATGCCACTCTTATCCATCAAT
CCATCACTGGTCTTTATGAAACACGCATTGATTTGAGTCAGCTAGGAGGTGACGGTGGAGGAGG
TTCTGGAGGTGGAGGTTCTGCTGAGTATGTGCGAGCCCTCTTTGACTTTAATGGGAATGATGAA
GAGGATCTTCCCTTTAAGAAAGGAGACATCCTGAGAATCCGGGATAAGCCTGAGGAGCAGTGGT
GGAATGCAGAGGACAGCGAAGGAAAGAGGGGGATGATTCCTGTCCCTTACGTGGAGAAGTATTC
CGGAGACTATAAGGACCACGACGGAGACTACAAGGATCATGATATTGATTACAAAGACGATGAC
GATAAGTCTAGGCTCGAGTAGgaggtctagaATGACCGACGCTGAGTACGTGAGAATCCATGAG
AAGTTGGACATCTACACGTTTAAGAAACAGTTTTTCAACAACAAAAAATCCGTGTCGCATAGAT
GCTACGTTCTCTTTGAATTAAAACGACGGGGTGAACGTAGAGCGTGTTTTTGGGGCTATGCTGT
GAATAAACCACAGAGCGGGACAGAACGTGGCATTCACGCCGAAATCTTTAGCATTAGAAAAGTC
GAAGAATACCTGCGCGACAACCCCGGACAATTCACGATAAATTGGTACTCATCCTGGAGTCCTT
GTGCAGATTGCGCTGAAAAGATCTTAGAATGGTATAACCAGGAGCTGCGGGGGAACGGCCACAC
TTTGAAAATCTGGGCTTGCAAACTCTATTACGAGAAAAATGCGAGGAATCAAATTGGGCTGTGG
AACCTCAGAGATAACGGGGTTGGGTTGAATGTAATGGTAAGTGAACACTACCAATGTTGCAGGA
AAATATTCATCCAATCGTCGCACAATCAATTGAATGAGAATAGATGGCTTGAGAAGACTTTGAA
GCGAGCTGAAAAACGACGGAGCGAGTTGTCCATTATGATTCAGGTAAAAATACTCCACACCACT
AAGAGTCCTGCTGTTGGCGGTGGTGGTAGTCCACCTCCAGCACTGCCTCCAAAGAGGAGGAGAT
GAatATGACCAACCTTTCCGACATCATAGAGAAGGAAACAGGCAAACAGTTGGTCATCCAAGAG
TCGATACTCATGCTTCCTGAAGAAGTTGAGGAGGTCATTGGGAATAAGCCGGAAAGTGACATTC
TCGTACACACTGCGTATGATGAGAGCACCGATGAGAACGTGATGCTGCTCACGTCAGATGCCCC
AGAGTACAAACCCTGGGCTCTGGTGATTCAGGACTCTAATGGAGAGAACAAGATCAAGATGCTA
GGACTGGTTGCATAAcggccgaagtcctgcagcattggattaattgttgacagtttcataactg
gctggtatattagtaaacgttgctttcaacataaattgacgatcattgtttaagagctatgctg
ttttgaatggtcccaaaacataaaacgaaaggctcagtcgaaagactgggcctttcgttttatc
actagtttaatcactttgactagcaaatactaacaacaagacacacacaccaaaaatcaaaaat
tcactactttagttaaaaaccacgtaaccacaagaactaatccaatccatgtaatcgggttct
tcaaatatttctccaagattttcctcctctaatatgctcaacttaaatgacctattcaataaat
ctattatgctgctaaatagtttataggacaaataagtatactctaatgacctataaaagataga
aaattaaaaaatcaagtgttcgcttcgctctcactgcccctcgacgttttagtagcctttccct
cacttcgttcagtccaagccaactaaaagttttcgggctactctctccttctcccctaataat
taattaaaatcttactctgtatatttctgctaatcattcgctaaacagcaaagaaaaaacaaac
acgtatcatagatataaatgtaatggcatagtgcgggttttatttTCAGCCTGTATCATAGCTA
AACAAATCGAGTTGTGTGTCCGTTTTAGGGCGTTCTGCTAGCTTGTTTAAAGTCTCTTGAATGA
ATGTATGCTCTAAGTCAAAAGAATTTGTCAGCGCCTTTATATAGCTTTCTTTTTCTTCTTTTTT
TACTTTAATGATCGATAGCAACAATGATTTAACACTAGCAAGTTGAATGCCACCATTTCTTCCT
GGTTTAATCTTAAAGAAAATTTCCTGATTCGCCTTCAGTACCTTCAGCAATTTATCTAATGTCC
GTTCAGGAATGCCTAGCACTTCTCTAATCTCTTTTTTGGTCGTCACTAAATAAGGCTTGTATAC

ATCGCTTTTTTCGCTAATATAAGCCATTAAATCTTCTTTCCATTCTGACAAATGAACACGTTGA
CGTTCGCTTCTTTTTTTCTTGAATTTAAACCACCCTTGACGGACAAATAAATCTTTACTGGTTA
AATCACTTGATACCCAAGCTTTGCAAAGAATGGTAATGTATTCCCTATTAGCCCCTTGATAGTT
TTCTGAATAGGCACTTCTAACAATTTTGATTACTTCTTTTTCTTCTAAGGGTTGATCTAATCGA
TTATTAAACTCAAACATATTATATTCGCACGTTTCGATTGAATAGCCTGAACTAAAGTAGGCTA
AAGAGAGGGTAAACATGACGTTATTACGCCCTATTAAACCCTTTTCTCCTGAAAATTTCGTTTC
GTGCAATAAGAGATTAAACCAGGGTTCATCTACTTGTTTTTTGCCTTCTGTACCGCTTAAAACC
GTTAGACTTGAACGAGTAAAGCCCTTATTATCTGTTTGTTTGAAAGACCAATCTTGCCATTCTT
TGAAAGAATAACGGTAATTAGGATCAAAAAATTCTACATTGTCCGTTCTTGGTATGCGAGCAAT
ACCAAAATGATTACACGTTAGATCAACTGGCAAAGACTTTCCAAAATATTCTCGGATATTTTGC
GAAATTATTTTGGCTGCTTTGACAGATTTAAATTCTGATTTTGAAGTCACATAGACTGGCGTTT
CTAAAACAAAATATGCTTGATAACCTTTATCAGATTTGATAATCATAGTAGGCATAAAACCTAA
ATCAATAGCGGTTGTTAAAATATCGCTTGCTGAAATAGTTTCTTTTGCCGTGTGAATATCAAAA
TCAATAAAGAAGGTATTGATTTGTCTTAAATTGTTTTCAGAATGTCCTTTCGTGTATGAACGGT
TTTCGTCTGCATACGTTCCATAACGATAAACGTTGGGTGTCCAATGTGTAAATGTATCTTGATT
TTCTTGAATCGCTTCCTCGGAAGTCAGAACAACACCACGACCGCCAATCATGCTTGATTTTGAG
CGATACGCAAAAATAGCCCCTTTGCTTTTACCTGGCTTGGTAGTGATTGAGCGAATTTTACTAT
TTTTAAATTTGTACTTTAACAAGCCGTCATGAAGCACAGTTTCTACAACAAAAGGATATTCAT
tcagctgttctcctttcctataaatcctataaaataggttgtTTAATTAACTTGGTTTGCTTTT
TCATTCAACTGTTTCAATATTGCATGTTTTGAAAAAGATTTTTTTCCTTTATAAGTCAATTTTT
TTCCACTAATCGAATAAATTATTTTGTTATTTTCTATTAACTTATATATATAATCTTCCCCCTC
CGAAGAAAAATACTTATCTGATTTTGTTTCTAAGTAGATATTTCTCTTTTCTAACTCTTTCTTA
AACGTTTCTAGTGTATAGATATTTGCTAATTTTCTTATCTCcaataaactatttttttatataag
ttttacattcatcatgattcatacaaactccaccttctataaatgaatacaaaaaaagcaatca
aacgatttccgattgattgcttaacaattcttaaattcagtagcttagatacttgaaaactctc
tgatttccctatataatgatagtacggttatataccgtcttcaaacaaagttaattaaataact
tcttacgagggaagagttcatctgactaactgataagcgttggtttggcaatcttatcgggcta
tgcatttataaaatgtcgtcaaacattttataaatgtgtcatggctctttttttcgtttctattc
agttcgttgtttcgttatatctagtataccgcttttaaaaaaaataagcaacgatttcgtgcat
tattcacacgaagtcattgctttttttcttcttccatttctaaatccaatgttacttgttctgat
tctgtttctggttctggttctgttggctcatttgggattaaatccactactagcgttgagttag
ttaactttgcaatttgttctagtgtttttatggttggatctgattttcctgattctattcgtga
ataatttgatctactcatttctaattcttgggtaccgccagcatttcggaaaaaaaccacgct
aaggatttttctataaaaagagccgttatattaagaataaaacggctcttttatacgtaaagg
acgtaaattcatttgcccagtgtcatgtaatccttcaaatttgtattctccaagaaaattgata
tgttcccatcctaacggccacgcatatggcattaaatcttctctaaattctcctcttgcttta
attcttctacggctttttccatatatacagtgttccacacacttatagcgttaataattatgtt
tagtgcactagctctttgtaactggtcttggagagcacgttctctaaattctccacgttgtcca
aaaaatataattctagctaatgcattgattgcttctcctttatttaaacctttttgaacccgtc
tccttacggctttattagatatgtaatccagcgtaaagagggttttctcgattcgtcccatttc
tccaagtgctgttgcgagtttattttgtcttgcatatgatccgagcttccccatgataagagcg
ctagggacctctttagctccttggaagctgtcagtagtatacctaataatttatctacattccc
tttagtaacgtgtaactttccaaatttacaaaagcgactcatagaaTTATTTCCTCCCGTTAAA
TAATAGATAACTATTAAAAATAGACAATACTTGCTCATAAGTAACGGTACTTAAATTGTTTACT
TTGGCGTGTTTCATTGCTTGATGAAACTGATTTTTAGTAAACAGTTGACGATATTCTCGATTGA
CCCATTTTGAAACAAAGTACGTATATAGCTTCCAATATTTATCTGGAACATCTGTGGTATGGCG
GGTAAGTTTATTAAGACACTGTTTACTTTTGGTTTAGGATGAAAGCATTCCGCTGGCAGCTTA

AGCAATTGCTGAATCGAGACTTGAGTGTGCAAGAGCAACCCTAGTGTTCGGTGAATATCCAAGG
TACGCTTGTAGAATCCTTCTTCAACAATCAGATAGATGTCAGACGCATGGCTTTCAAAAACCAC
TTTTTTAATAATTTGTGTGCTTAAATGGTAAGGAATACTCCCAACAATTTTATACCTCTGTTTG
TTAGGGAATTGAAACTGTAGAATATCTTGGTGAATTAAAGTGACACGAGTATTCAGTTTTAATT
TTTCTGACGATAAGTTGAATAGATGACTGTCTAATTCAATAGACGTTACCTGTTTACTTATTTT
AGCCAGTTTCGTCGTTAAATGCCCTTTACCTGTTCCAATTTCGTAAACGGTATCGGTTTCTTTT
AAATTCAATTGTTTTATTATTTGGTTGAGTACTTTTTCACTCGTTAAAAAGTTTTGAGAATATT
TTATATTTTTGTTCATgtaatcactccttcttaattacaaatttttagcatctaatttaacttc
aattcctattatacaaaattttaagatactgcactatcaacacactcttaagtttgcttctaag
tcttatttccataacttcttttacgtttccgccattctttgctgtttcgatttttatgatatgg
tgcaagtcagcacgaacacgaaccgtcttatctcccattatatctttttttgcactgattggtg
tatcatttcgtttttctttttgtgcgcttcttgataaaagggatagtaattcattcctggttgc
aaatttgaaaaccgctacggatcgcatctttttctaaactagggcccacaacggcgctgccga
cctgggtcagcatcaatttccataggctccgcccccctgacgagcatcacaaaaatcgacgctc
aagtcagaggtggcgaaacccgacaggactataaagataccaggcgtttccccctggaagctcc
ctcgtgcgctctcctgttccgaccctgccgcttaccggatacctgtccgcctttctcccttcgg
gaagcgtggcgctttctcatagctcacgctgtaggtatctcagttcggtgtaggtcgttcgctc
caagctgggctgtgtgcacgaaccccccgttcagcccgaccgctgcgccttatccggtaactat
cgtcttgagtccaacccggtaagacacgacttatcgccactggcagcagccactggtaacagga
ttagcagagcgaggtatgtaggcggtgctacagagttcttgaagtggtggcctaactacggcta
cactagaagaacagtatttggtatctgcgctctgctgaagccagttaccttcggaaaaagagtt
ggtagctcttgatccggcaaacaaaccaccgctggtagcggtggtttttttgtttgcaagcagc
agattacgcgcagaaaaaaaggatctcaagaagatcctttgatcttttctacggggtctgacgc
tcagtggaacgaaaactcacgttaagggattttggtcatgagattatcaaaaaggatcttcacc
tagatccttttaaattaaaaatgaagttttaaatcaatctaaagtatatatgagtaaacttggt
ctgacagTTAGAAAAACTCATCGAGCATCAAATGAAACTGCAATTTATTCATATCAGGATTATC
AATACCATATTTTTGAAAAAGCCGTTTCTGTAATGAAGGAGAAAACTCACCGAGGCAGTTCCAT
AGGATGGCAAGATCCTGGTATCGGTCTGCGATTCCGACTCGTCCAACATCAATACAACCTATTA
ATTTCCCCTCGTCAAAAATAAGGTTATCAAGTGAGAAATCACCATGAGTGACGACTGAATCCGG
TGAGAATGGCAAAAGTTTATGCATTTCTTTCCAGACTTGTTCAACAGGCCAGCCATTACGCTCG
TCATCAAAATCACTCGCATCAACCAAACCGTTATTCATTCGTGATTGCGCCTGAGCGAGACGAA
ATACGCGGTCGCTGTTAAAAGGACAATTACAAACAGGAATCGAATGCAACCGGCGCAGGAACAC
TGCCAGCGCATCAACAATATTTTCACCTGAATCAGGATATTCTTCTAATACCTGGAATGCTGTT
TTCCCAGGGATCGCAGTGGTGAGTAACCATGCATCATCAGGAGTACGGATAAAATGCTTGATGG
TCGGAAGAGGCATAAATTCCGTCAGCCAGTTTAGTCTGACCATCTCATCTGTAACATCATTGGC
AACGCTACCTTTGCCATGTTTCAGAAACAACTCTGGCGCATCGGGCTTCCCATACAATCGATAG
ATTGTCGCACCTGATTGCCCGACATTATCGCGAGCCCATTTATACCCATATAAATCAGCATCCA
TGTTGGAATTTAATCGCGGCCTAGAGCAAGACGTTTCCCGTTGAATATGGCTCATactcttcct
ttttcaatattattgaagcatttatcagggttattgtctcatgagcggatacatatttgaatgt
atttagaaaaataaacaaataggggttccgcgaccggttatcg

Supplementary Note 4

Distinct elements from pYK01 and pYK06 in the other vectors

Prrn4b (Lp)

attggattaattgttgacagtttcataactggctggtatattagtaaacgttgct

Prrn3a (Lp)

ttataaaaagatgttgacagcttgttctgatgatgataaactttaatagttgcg

rrnA (Ls) terminator for tracrRNA

aaaaaggttgaaccttagggttcagccttttt

rrnB T1 (Ec) terminator for crRNA

ataaaacgaaaggctcagtcgaaagactgggcctttcgttttat

dCas9

ATGGATAAGAAATACTCAATAGGCTTAGCTATCGGCACAAATAGCGTCGGATGGGCGGTGATCA
CTGATGAATATAAGGTTCCGTCTAAAAAGTTCAAGGTTCTGGGAAATACAGACCGCCACAGTAT
CAAAAAAAATCTTATAGGGGCTCTTTTATTTGACAGTGGAGAGACAGCGGAAGCGACTCGTCTC
AAACGGACAGCTCGTAGAAGGTATACACGTCGGAAGAATCGTATTTGTTATCTACAGGAGATTT
TTTCAAATGAGATGGCGAAAGTAGATGATAGTTTCTTTCATCGACTTGAAGAGTCTTTTTTGGT
GGAAGAAGACAAGAAGCATGAACGTCATCCTATTTTTGGAAATATAGTAGATGAAGTTGCTTAT
CATGAGAAATATCCAACTATCTATCATCTGCGAAAAAAATTGGTAGATTCTACTGATAAAGCGG
ATTTGCGCTTAATCTATTTGGCCTTAGCGCATATGATTAAGTTTCGTGGTCATTTTTTGATTGA
GGGAGATTTAAATCCTGATAATAGTGATGTGGACAAACTATTTATCCAGTTGGTACAAACCTAC
AATCAATTATTTGAAGAAAACCCTATTAACGCAAGTGGAGTAGATGCTAAAGCGATTCTTTCTG
CACGATTGAGTAAATCAAGACGATTAGAAAATCTCATTGCTCAGCTCCCCGGTGAGAAGAAAAA
TGGCTTATTTGGGAATCTCATTGCTTTGTCATTGGGTTTGACCCCTAATTTTAAATCAAATTTT
GATTTGGCAGAAGATGCTAAATTACAGCTTTCAAAAGATACTTACGATGATGATTTAGATAATT
TATTGGCGCAAATTGGAGATCAATATGCTGATTTGTTTTTGGCAGCTAAGAATTTATCAGATGC
TATTTTACTTTCAGATATCCTAAGAGTAAATACTGAAATAACTAAGGCTCCCCTATCAGCTTCA
ATGATTAAACGCTACGATGAACATCATCAAGACTTGACTCTTTTAAAAGCTTTAGTTCGACAAC
AACTTCCAGAAAAGTATAAAGAAATCTTTTTTGATCAATCAAAAAACGGATATGCAGGTTATAT
TGATGGGGGAGCTAGCCAAGAAGAATTTTATAAATTTATCAAACCAATTTTAGAAAAAATGGAT
GGTACTGAGGAATTATTGGTGAAACTAAATCGTGAAGATTTGCTGCGCAAGCAACGGACCTTTG
ACAACGGCTCTATTCCCCATCAAATTCACTTGGGTGAGCTGCATGCTATTTTGAGAAGACAAGA
AGACTTTTATCCATTTTTAAAAGACAATCGTGAGAAGATTGAAAAAATCTTGACTTTTCGAATT
CCTTATTATGTTGGTCCATTGGCGCGTGGCAATAGTCGTTTTGCATGGATGACTCGGAAGTCTG
AAGAAACAATTACCCCATGGAATTTTGAAGAAGTTGTCGATAAAGGTGCTTCAGCTCAATCATT
TATTGAACGCATGACAAACTTTGATAAAAATCTTCCAAATGAAAAAGTACTACCAAAACATAGT
TTGCTTTATGAGTATTTTACGGTTTATAACGAATTGACAAAGGTCAAATATGTTACTGAAGGAA

TGCGAAAACCAGCATTTCTTTCAGGTGAACAGAAGAAAGCCATTGTTGATTTACTCTTCAAAAC
AAATCGAAAAGTAACCGTTAAGCAATTAAAAGAAGATTATTTCAAAAAAATAGAATGTTTTGAT
AGTGTTGAAATTTCAGGAGTTGAAGATAGATTTAATGCTTCATTAGGTACCTACCATGATTTGC
TAAAAATTATTAAAGATAAAGATTTTTTGGATAATGAAGAAAATGAAGATATCTTAGAGGATAT
TGTTTTAACATTGACCTTATTTGAAGATAGGGAGATGATTGAGGAAAGACTTAAAACATATGCT
CACCTCTTTGATGATAAGGTGATGAAACAGCTTAAACGTCGCCGTTATACTGGTTGGGGACGTT
TGTCTCGAAAATTGATTAATGGTATTAGGGATAAGCAATCTGGCAAAACAATATTAGATTTTTT
GAAATCAGATGGTTTTGCCAATCGCAATTTTATGCAGCTGATCCATGATGATAGTTTGACATTT
AAAGAAGACATTCAAAAAGCACAAGTGTCTGGACAAGGCGATAGTTTACATGAACATATTGCAA
ATTTAGCTGGTAGCCCTGCTATTAAAAAAGGTATTTTACAGACTGTAAAAGTTGTTGATGAATT
GGTCAAAGTAATGGGGCGGCATAAGCCAGAAAATATCGTTATTGAAATGGCACGTGAAAATCAG
ACAACTCAAAAGGGCCAGAAAAATTCGCGAGAGCGTATGAAACGAATCGAAGAAGGTATCAAAG
AATTAGGAAGTCAGATTCTTAAAGAGCATCCTGTTGAAAATACTCAATTGCAAAATGAAAAGCT
CTATCTCTATTATCTCCAAAATGGAAGAGACATGTATGTGGACCAAGAATTAGATATTAATCGT
TTAAGTGATTATGATGTCGATGCCATTGTTCCACAAAGTTTCCTTAAAGACGATTCAATAGACA
ATAAGGTCTTAACGCGTTCTGATAAAAATCGTGGTAAATCGGATAACGTTCCAAGTGAAGAAGT
AGTCAAAAAGATGAAAAACTATTGGAGACAACTTCTAAACGCCAAGTTAATCACTCAACGTAAG
TTTGATAATTTAACGAAAGCTGAACGTGGAGGTTTGAGTGAACTTGATAAAGCTGGTTTTATCA
AACGCCAATTGGTTGAAACTCGCCAAATCACTAAGCATGTGGCACAAATTTTGGATAGTCGCAT
GAATACTAAATACGATGAAAATGATAAACTTATTCGAGAGGTTAAAGTGATTACCTTAAAATCT
AAATTAGTTTCTGACTTCCGAAAAGATTTCCAATTCTATAAAGTACGTGAGATTAACAATTACC
ATCATGCCCATGATGCGTATCTAAATGCCGTCGTTGGAACTGCTTTGATTAAGAAATATCCAAA
ACTTGAATCGGAGTTTGTCTATGGTGATTATAAAGTTTATGATGTTCGTAAAATGATTGCTAAG
TCTGAGCAAGAAATAGGCAAAGCAACCGCAAAATATTTCTTTTACTCTAATATCATGAACTTCT
TCAAAACAGAAATTACACTTGCAAATGGAGAGATTCGCAAACGCCCTCTAATCGAAACTAATGG
GGAAACTGGAGAAATTGTCTGGGATAAAGGGCGAGATTTTGCCACAGTGCGCAAAGTATTGTCC
ATGCCCCAAGTCAATATTGTCAAGAAAACAGAAGTACAGACAGGCGGATTCTCCAAGGAGTCAA
TTTTACCAAAAAGAAATTCGGACAAGCTTATTGCTCGTAAAAAAGACTGGGATCCAAAAAAATA
TGGTGGTTTTGATAGTCCAACGGTAGCTTATTCAGTCCTAGTGGTTGCTAAGGTGGAAAAAGGG
AAATCGAAGAAGTTAAAATCCGTTAAAGAGTTACTAGGGATCACAATTATGGAAAGAAGTTCCT
TTGAAAAAAATCCGATTGACTTTTTAGAAGCTAAAGGATATAAGGAAGTTAAAAAAGACTTAAT
CATTAAACTACCTAAATATAGTCTTTTTGAGTTAGAAAACGGTCGTAAACGGATGCTGGCTAGT
GCCGGAGAATTACAAAAAGGAAATGAGCTGGCTCTGCCAAGCAAATATGTGAATTTTTTATATT
TAGCTAGTCATTATGAAAAGTTGAAGGGTAGTCCAGAAGATAACGAACAAAAACAATTGTTTGT
GGAGCAGCATAAGCATTATTTAGATGAGATTATTGAGCAAATCAGTGAATTTTCTAAGCGTGTT
ATTTTAGCAGATGCCAATTTAGATAAAGTTCTTAGTGCATATAACAAACATAGAGACAAACCAA
TACGTGAACAAGCAGAAAATATTATTCATTTATTTACGTTGACGAATCTTGGAGCTCCCGCTGC
TTTTAAATATTTTGATACAACAATTGATCGTAAACGATATACGTCTACAAAAGAAGTTTTAGAT
GCCACTCTTATCCATCAATCCATCACTGGTCTTTATGAAACACGCATTGATTTGAGTCAGCTAG
GAGGTGAC

NG-nCas9

ATGGACAAGAAGTACAGCATCGGCCTGGCCATCGGCACCAACTCTGTGGGCTGGGCCGTGATCA
CCGACGAGTACAAGGTGCCCAGCAAGAAATTCAAGGTGCTGGGCAACACCGACCGGCACAGCAT
CAAGAAGAACCTGATCGGAGCCCTGCTGTTCGACAGCGGCGAAACAGCCGAGGCCACCCGGCTG
AAGAGAACCGCCAGAAGAAGATACACCAGACGGAAGAACCGGATCTGCTATCTGCAAGAGATCT

TCAGCAACGAGATGGCCAAGGTGGACGACAGCTTCTTCCACAGACTGGAAGAGTCCTTCCTGGT
GGAAGAGGATAAGAAGCACGAGCGGCACCCCATCTTCGGCAACATCGTGGACGAGGTGGCCTAC
CACGAGAAGTACCCCACCATCTACCACCTGAGAAAGAAACTGGTGGACAGCACCGACAAGGCCG
ACCTGCGGCTGATCTATCTGGCCCTGGCCCACATGATCAAGTTCCGGGGCCACTTCCTGATCGA
GGGCGACCTGAACCCCGACAACAGCGACGTGGACAAGCTGTTCATCCAGCTGGTGCAGACCTAC
AACCAGCTGTTCGAGGAAAACCCCATCAACGCCAGCGGCGTGGACGCCAAGGCCATCCTGTCTG
CCAGACTGAGCAAGAGCAGACGGCTGGAAAATCTGATCGCCCAGCTGCCCGGCGAGAAGAAGAA
TGGCCTGTTCGGAAACCTGATTGCCCTGAGCCTGGGCCTGACCCCCAACTTCAAGAGCAACTTC
GACCTGGCCGAGGATGCCAAACTGCAGCTGAGCAAGGACACCTACGACGACGACCTGGACAACC
TGCTGGCCCAGATCGGCGACCAGTACGCCGACCTGTTTCTGGCCGCCAAGAACCTGTCCGACGC
CATCCTGCTGAGCGACATCCTGAGAGTGAACACCGAGATCACCAAGGCCCCCCTGAGCGCCTCT
ATGATCAAGAGATACGACGAGCACCACCAGGACCTGACCCTGCTGAAAGCTCTCGTGCGGCAGC
AGCTGCCTGAGAAGTACAAAGAGATTTTCTTCGACCAGAGCAAGAACGGCTACGCCGGCTACAT
TGACGGCGGAGCCAGCCAGGAAGAGTTCTACAAGTTCATCAAGCCCATCCTGGAAAAGATGGAC
GGCACCGAGGAACTGCTCGTGAAGCTGAACAGAGAGGACCTGCTGCGGAAGCAGCGGACCTTCG
ACAACGGCAGCATCCCCCACCAGATCCACCTGGGAGAGCTGCACGCCATTCTGCGGCGGCAGGA
AGATTTTTACCCATTCCTGAAGGACAACCGGGAAAAGATCGAGAAGATCCTGACCTTCCGCATC
CCCTACTACGTGGGCCCTCTGGCCAGGGGAAACAGCAGATTCGCCTGGATGACCAGAAAGAGCG
AGGAAACCATCACCCCCTGGAACTTCGAGGAAGTGGTGGACAAGGGCGCTTCCGCCCAGAGCTT
CATCGAGCGGATGACCAACTTCGATAAGAACCTGCCCAACGAGAAGGTGCTGCCCAAGCACAGC
CTGCTGTACGAGTACTTCACCGTGTATAACGAGCTGACCAAAGTGAAATACGTGACCGAGGGAA
TGAGAAAGCCCGCCTTCCTGAGCGGCGAGCAGAAAAAGGCCATCGTGGACCTGCTGTTCAAGAC
CAACCGGAAAGTGACCGTGAAGCAGCTGAAAGAGGACTACTTCAAGAAAATCGAGTGCTTCGAC
TCCGTGGAAATCTCCGGCGTGGAAGATCGGTTCAACGCCTCCCTGGGCACATACCACGATCTGC
TGAAAATTATCAAGGACAAGGACTTCCTGGACAATGAGGAAAACGAGGACATTCTGGAAGATAT
CGTGCTGACCCTGACACTGTTTGAGGACAGAGAGATGATCGAGGAACGGCTGAAAACCTATGCC
CACCTGTTCGACGACAAAGTGATGAAGCAGCTGAAGCGGCGGAGATACACCGGCTGGGGCAGGC
TGAGCCGGAAGCTGATCAACGGCATCCGGGACAAGCAGTCCGGCAAGACAATCCTGGATTTCCT
GAAGTCCGACGGCTTCGCCAACAGAAACTTCATGCAGCTGATCCACGACGACAGCCTGACCTTT
AAAGAGGACATCCAGAAAGCCCAGGTGTCCGGCCAGGGCGATAGCCTGCACGAGCACATTGCCA
ATCTGGCCGGCAGCCCCGCCATTAAGAAGGGCATCCTGCAGACAGTGAAGGTGGTGGACGAGCT
CGTGAAAGTGATGGGCCGGCACAAGCCCGAGAACATCGTGATCGAAATGGCCAGAGAGAACCAG
ACCACCCAGAAGGGACAGAAGAACAGCCGCGAGAGAATGAAGCGGATCGAAGAGGGCATCAAAG
AGCTGGGCAGCCAGATCCTGAAAGAACACCCCGTGGAAAACACCCAGCTGCAGAACGAGAAGCT
GTACCTGTACTACCTGCAGAATGGGCGGGATATGTACGTGGACCAGGAACTGGACATCAACCGG
CTGTCCGACTACGATGTGGACCATATCGTGCCTCAGAGCTTTCTGAAGGACGACTCCATCGACA
ACAAGGTGCTGACCAGAAGCGACAAGAACCGGGGCAAGAGCGACAACGTGCCCTCCGAAGAGGT
CGTGAAGAAGATGAAGAACTACTGGCGGCAGCTGCTGAACGCCAAGCTGATTACCCAGAGAAAG
TTCGACAATCTGACCAAGGCCGAGAGAGGCGGCCTGAGCGAACTGGATAAGGCCGGCTTCATCA
AGAGACAGCTGGTGGAAACCCGGCAGATCACAAAGCACGTGGCACAGATCCTGGACTCCCGGAT
GAACACTAAGTACGACGAGAATGACAAGCTGATCCGGGAAGTGAAAGTGATCACCCTGAAGTCC
AAGCTGGTGTCCGATTTCCGGAAGGATTTCCAGTTTTACAAAGTGCGCGAGATCAACAACTACC
ACCACGCCCACGACGCCTACCTGAACGCCGTCGTGGGAACCGCCCTGATCAAAAAGTACCCTAA
GCTGGAAAGCGAGTTCGTGTACGGCGACTACAAGGTGTACGACGTGCGGAAGATGATCGCCAAG
AGCGAGCAGGAAATCGGCAAGGCTACCGCCAAGTACTTCTTCTACAGCAACATCATGAACTTTT
TCAAGACCGAGATTACCCTGGCCAACGGCGAGATCCGGAAGCGGCCTCTGATCGAGACAAACGG
CGAAACCGGGGAGATCGTGTGGGATAAGGGCCGGGATTTTGCCACCGTGCGGAAAGTGCTGAGC

ATGCCCCAAGTGAATATCGTGAAAAAGACCGAGGTGCAGACAGGCGGCTTCAGCAAAGAGTCTA
TCCGGCCCAAGAGGAACAGCGATAAGCTGATCGCCAGAAAGAAGGACTGGGACCCTAAGAAGTA
CGGCGGCTTCGTGAGCCCCACCGTGGCCTATTCTGTGCTGGTGGTGGCCAAAGTGGAAAAGGGC
AAGTCCAAGAAACTGAAGAGTGTGAAAGAGCTGCTGGGGATCACCATCATGGAAAGAAGCAGCT
TCGAGAAGAATCCCATCGACTTTCTGGAAGCCAAGGGCTACAAAGAAGTGAAAAAGGACCTGAT
CATCAAGCTGCCTAAGTACTCCCTGTTCGAGCTGGAAAACGGCCGGAAGAGAATGCTGGCCTCT
GCCCGGTTCCTGCAGAAGGGAAACGAACTGGCCCTGCCCTCCAAATATGTGAACTTCCTGTACC
TGGCCAGCCACTATGAGAAGCTGAAGGGCTCCCCCGAGGATAATGAGCAGAAACAGCTGTTTGT
GGAACAGCACAAGCACTACCTGGACGAGATCATCGAGCAGATCAGCGAGTTCTCCAAGAGAGTG
ATCCTGGCCGACGCTAATCTGGACAAAGTGCTGTCCGCCTACAACAAGCACCGGGATAAGCCCA
TCAGAGAGCAGGCCGAGAATATCATCCACCTGTTTACCCTGACCAATCTGGGAGCCCCTCGGGC
CTTCAAGTACTTTGACACCACCATCGACCGGAAGGTGTACCGGAGCACCAAAGAGGTGCTGGAC
GCCACCCTGATCCACCAGAGCATCACCGGCCTGTACGAGACACGGATCGACCTGTCTCAGCTGG
GAGGTGAC
